# The initial expression alterations occurring to transcription factors during the formation of breast cancer: Evidence from bioinformatics

**DOI:** 10.1002/cam4.4545

**Published:** 2022-01-17

**Authors:** Xingxing Dong, Yalong Yang, Gaoran Xu, Zelin Tian, Qian Yang, Yan Gong, Gaosong Wu

**Affiliations:** ^1^ Department of Thyroid and Breast Surgery Zhongnan Hospital of Wuhan University Wuhan China; ^2^ Tumor Precision Diagnosis and Treatment Technology and Translational Medicine Hubei Engineering Research Center Zhongnan Hospital of Wuhan University Wuhan China; ^3^ Department of Biological Repositories Zhongnan Hospital of Wuhan University Wuhan China

**Keywords:** bioinformatics, breast cancer, diagnosis, oncogenesis, prognosis

## Abstract

**Background:**

Breast cancer (BC) is the leading malignancy among women worldwide.

**Aim:**

This work aimed to present a comprehensively bioinformatic analysis of gene expression profiles and to identify the hub genes during BC tumorigenesis, providing potential biomarkers and targets for the diagnosis and therapy of BC.

**Materials & Methods:**

In this study, multiple public databases, bioinformatics approaches, and online analytical tools were employed and the real‐time reverse transcription polymerase chain reaction was implemented.

**Results:**

First, we identified 10, 107, and 3869 differentially expressed genes (DEGs) from three gene expression datasets (GSE9574, GSE15852, and GSE42568, covering normal, para‐cancerous, and BC samples, respectively), and investigated different biological functions and pathways involved. Then, we screened out 8, 16, and 29 module genes from these DEGs, respectively. Next, 10 candidate genes were determined through expression and survival analyses. We noted that seven candidate genes *JUN*, *FOS*, *FOSB*, *EGR1*, *ZFP36*, *CFD*, and *PPARG* were downregulated in BC compared to normal tissues and lower expressed in aggressive types of BC (basal, HER2^+^, and luminal B), *TP53* mutation group, younger patients, higher stage BC, and lymph node metastasis BC, while *CD27*, *PSMB9*, and *SELL* were upregulated. The present study discovered that the expression levels of these candidate genes were correlated with the infiltration of immune cells (CD8^+^ T cell, macrophage, natural killer [NK] cell, and cancer‐associated fibroblast) in BC, as well as biomarkers of immune cells and immune checkpoints. We also revealed that promoter methylation, amplification, and deep deletion might contribute to the abnormal expressions of candidate genes. Moreover, we illustrated downstream‐targeted genes of *JUN*, *FOS*, *FOSB*, *EGR1*, and *ZFP36* and demonstrated that these targeted genes were involved in “positive regulation of cell death”, “pathways in cancer”, “PI3K‐Akt signaling pathway”, and so on.

**Discussion & Conclusion:**

We presented differential gene expression profiles among normal, para‐cancerous, and BC tissues and further identified candidate genes that might contribute to tumorigenesis and progression of BC, as potential diagnostic and prognostic targets for BC patients.

## Graphical abstract

In this work, we focused on the transcriptional aberrations during the formation of breast cancer (BC) and explored the molecular mechanisms that might be relevant using bioinformatic approaches, and found the initial expression alterations of BC might occur to transcription factors. Our findings provide novel insights in gene expression during BC tumorigenesis and promising biomarkers for early diagnosis, prevention of metastasis, and targeted therapies in BC.

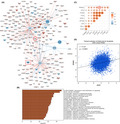

## 1 INTRODUCTION

Breast cancer (BC) is currently the most commonly diagnosed cancer according to Global Cancer Statistics 2020 covering 36 cancers in 185 countries, the new cases of which were up to 2,261,419, and the new deaths were 684,996.^1^ Women are the main sufferers with the high incidence and mortality, obviously increasing female extra cancer burden. According to the morphological, molecular, and genetic heterogeneities,^2^ BC is divided into four major subtypes with the expression of markers (estrogen receptor, ER; progesterone receptor, PR; human epidermal growth factor receptor 2, HER2): luminal A (ER^+^ and/or PR^+^, HER2^−^, Ki‐67^−^), luminal B (ER^+^ and/or PR^+^, HER2^+^, Ki‐67^+^), HER2^+^ (ER^−^ and PR^−^, HER2^+^), and basal or triple‐negative (ER^−^, PR^−^ and HER2^−^).^3^ Numerous studies proved that therapeutic approaches and patients' outcomes of BC varied largely based on cancer stages and subtypes at diagnosis, as well as ages, tumor grades, and lymphovascular status.^4^ The 5‐year overall survival (OS) rates for stage I and II patients were over 90%, receiving surgery with or without adjuvant radiation and chemotherapy. However, it was less than 30% for stage IV patients treated with palliative/noncurative‐intent therapy or radiation/chemotherapy alone. Compared with ER^+^/PR^+^ and HER2^+^ BC patients benefiting from endocrine and targeted therapies with chemotherapy, triple‐negative, or basal BC patients have poorer prognoses due to limited treatment options.^4^, ^5^, ^6^ In the last decades, PD‐L1 inhibition has been proved to ameliorate the progression‐free survival (PFS) of triple‐negative BC (TNBC) patients.^7^ However, the mechanisms of carcinogenesis and progression of BC are still ambiguous, which need to be further elucidated. More effectively therapeutic targets are as well essential to be explored.

In the last two decades, high‐throughput technologies and next‐generation sequencing^8^ have developed rapidly, as well as bioinformatics,^9^ which make it possible to deeply explore the etiology, pathogenic mechanism, progress, treatment of diseases at the genetic level. Moreover, public databases, data processing software, and online tools^10^, ^11^ make it convenient for researchers to brainstorm all over the world. Nevertheless, the common practices are to identify differentially expressed genes (DEGs) between normal and aberrant tissues as potential biomarkers for diagnosis, prognosis, or therapeutic targets of diseases with bioinformatic approaches.^12^, ^13^, ^14^, ^15^, ^16^, ^17^ In this study, we also investigated the differences between para‐cancerous (histologically normal) tissues from BC patients and normal tissues from cancer‐free patients in addition to the well‐studied changes between histologically normal and cancer tissues, attempting to reveal the main disturbances and any subtle clues from normal breast tissue to BC based on differential gene expression profiles among them. And further to identify candidate genes that contribute to tumorigenesis and progression of BC, and are associated with the prognosis of BC patients as potential diagnostic and therapeutic targets.

First, we screened and selected three gene expression datasets GSE9574 (containing normal and para‐cancerous samples), GSE15852 (containing para‐cancerous and BC samples), and GSE42568 (containing normal and BC samples) from Gene Expression Omnibus (GEO) database, and identified DEGs from them, respectively. Then, the corresponding functions and pathways enriched by these DEGs were explored using the Gene Ontology (GO) function and Kyoto Encyclopedia of Genes and Genomes (KEGG) pathway enrichment analyses. The corresponding module genes were screened out from these DEGs based on networks and module analysis. Next, the candidate genes were determined through expression validations of these module genes with The Cancer Genome Atlas (TCGA) and survival analysis. Subsequently, we evaluated expression levels of these candidate genes based on clinical features and *TP53* mutation status, and also investigated the correlations between their expression and immune cell infiltration in BC, as well as biomarkers of immune cells and immune checkpoints. Moreover, we estimated genetic alteration and methylation of candidate genes and explored targeted genes of transcription factors (TFs) in them. More convincingly, we further assessed candidate gene expression levels in clinical samples. In summary, our work implemented a comprehensive bioinformatic analysis on BC, showing different transcriptional variations in para‐cancerous and BC tissues, and enriching our understanding of the mechanisms of BC tumorigenesis, invasion, relapse, progression, and prognosis. Our results provided promisingly diagnostic and therapeutic targets for BC patients.

## 2 MATERIALS AND METHODS

### 2.1 Raw data acquisition

Three gene expression datasets (GSE9574, GSE15852, and GSE42568) of BC were screened and selected from GEO (https://www.ncbi.nlm.nih.gov/geo/),^18^ including raw CEL files and their corresponding platform annotation files (i.e., GPL files). In dataset GSE9574 based on GPL96 platforms ([HG‐U133A] Affymetrix Human Genome U133A Array), 29 samples were obtained from histologically normal breast epithelium, of which 14 samples were from epithelium adjacent to a breast tumor, and 15 samples were from patients undergoing reduction mammoplasty without apparent BC.^19^ In dataset GSE15852 also based on GPL96 Platforms, 86 sets of gene expression data were obtained from 43 pairs of tumors and adjacent‐tumor tissues.^20^ The GSE42568 dataset was based on GPL570 Platforms ([HG‐U133_Plus_2] Affymetrix Human Genome U133 Plus 2.0 Array) and contained 104 primary BC and 17 normal breast biopsies gene chips.^21^

### 2.2 Raw data quality control

The normalized unscaled standard error (NUSE) plots^22^ were selected to test the consistency of the data, and RNA degradation plots were to estimate the RNA degradation of the microarray gene chips with affyPLM and affy packages in R (version 4.0.3). For box charts of NUSE, the median standard error across gene chips close to 1 and not higher than 1.05 indicated good consistency. The chips not meeting the criteria were omitted to assure the data quality. As for RNA degradation plots, in a general way, much higher slope and poor uniformity with other chips, especially the latter, indicated that their RNA degradation was outrageous and substantially impacts data quality.^22^

### 2.3 Raw data preprocessing

The log scale robust multi‐array analysis^23^ was applied to transform probe intensity data of gene chips stored in CEL files into expression values, including three main steps: background correction, normalization, and log2 transformation of PM values, through rma function of R package affy. Probe IDs were then turned into gene symbols by R with the expression files of probe set and their relevant GPL files. The mean of their expression values was used to avoid more than one probe corresponding to one gene. Finally, K Nearest Neighbors algorithm^24^ was used to estimate and impute missing value of gene expression matrix carried out by R package impute after the rows without gene symbols were deleted.

### 2.4 DEG identification

The limma^25^ package of R was used to identify DEGs from processed data according to the cutoff criterion of adjusted p value <0.05 and log2(fold change) > 1 or log2(fold change) < −1. To visualize the discrepancy, we invoked ggpubr package based on ggplot2 and ggthemes package of R to draw volcano plots. Furthermore, the Venn diagram was portrayed using an online tool (http://bioinformatics.psb.ugent.be/webtools/Venn/) to observe the relationships among three DEGs groups clearly. Meanwhile, the concrete overlapping and nonoverlapping genes among them were also obtained.

### 2.5 GO function and KEGG pathway enrichment analyses of DEGs

The GO function^26^, ^27^ and KEGG pathway^28^ enrichment analyses of DEGs were accomplished using R packages org. Hs.eg.db, AnnotationDbi, and clusterProfiler with adjusted *p* value cutoff = 0.05 and merged terms of biological process in GO by the cutoff = 0.7. Furthermore, bubble diagrams were drawn using R packages to make the results more intuitionistic.

### 2.6 Network construction and module analysis

The STRING online database (version 11, http://string‐db.org/)^29^ was used to construct protein‐protein interaction (PPI) networks of DEGs identified from GSE9574 and GSE15852 datasets with minimum required interaction score: medium confidence (0.400) and applied TSV files containing the detail information of the networks. The weighted gene co‐expression network analysis (WGCNA)^30^ was performed to obtain the detail information of the gene co‐expression network for DEGs from GSE42568 dataset through the corresponding R package WGCNA^31^ (version 1.70‐3) with signed *R* ^2^ > 0.85. Cytoscape (version 3.8.2)^32^ was then employed to reproduce PPI networks via importing the TSV files and building a gene co‐expression network. Subsequently, plug‐in MCODE^33^ of Cytoscape was used to detect the densely connected regions (i.e., modules) of networks, the criteria of which were a degree cutoff = 2, MCODE scores ≥ 4, Max. Depth = 100, *k*‐score = 2, and node score cutoff = 0.2. The online tool Metascape (https://metascape.org/gp/index.html#/main/step1)^34^ was applied to conduct functional enrichment analysis of module DEGs, and the R package clusterProfiler (version 3.16.1) was applied to explore cellular component terms of GO and KEGG pathways enriched by these module genes with adjusted *p* value cutoff = 0.05.

### 2.7 TF analysis

Transcription factor information was downloaded from Transcriptional Regulatory Relationships Unraveled by Sentence‐based Text mining^35^ (TRRUST; www.grnpedia.org/trrust ). The online tool Draw Venn Diagram was applied to identify TFs from module genes screened out from the three datasets.

### 2.8 Expression validation and survival analysis

All identified module genes were validated with UALCAN^36^ (http://ualcan.path.uab.edu/) among a total of 114 normal and 1097 primary breast tumor samples based on TCGA,^37^ with a threshold of *p* < 0.05. For validated genes, the Breast Cancer Gene‐Expression Miner v4.7^38^ (bc‐GenExMiner v4.7; http://bcgenex.ico.unicancer.fr/BC‐GEM/GEM‐Accueil.php?js=1) were used for survival analysis based on DNA microarrays (*n* = 11,359) in “PROGNOSIS” of “ANALYSIS Module”. Clearly, the OS for all module genes was primarily performed among all BC patients and then for genes whose expression levels were significantly associated with OS, and the disease‐free survival (DFS) and distant metastasis‐free survival (DMFS) analysis were performed. Next, we downloaded TCGA dataset of BC, consisting of gene expression RNAseq data (*n* = 1218), clinical phenotype data (*n* = 1247), curated survival data (*n* = 1236), and somatic mutation data (MC3 gene‐level non‐silent mutation, *n* = 791) from UCSC Xena web (https://xenabrowser.net/DATAPAGES/) for the univariate and multivariate OS analysis of genes associated with OS above using Cox Proportional Hazards model carried out by R package survival. Briefly, we first applied separate univariate Cox regressions to assess the statistical significance for each gene, BC patient stage, age, ER/PR/HER2 status, molecular subtype, tumor/node/metastasis (TNM) status, and *TP53* mutation status in relation to OS, then performed multivariate Cox regression analysis for each significant gene combining with all the variables. Expression analysis based on BC patients’ clinical phenotype and *TP53* status.

Subsequently, the bc‐GenExMiner v4.7 resource^39^ was applied to explore candidate gene expression levels with DNA microarrays data (*n* = 11,359) in the “EXPRESSION” of “ANALYSIS Module” based on intrinsic molecular subtypes (PAM50 subtypes), patient's ages, nodal metastasis statuses, and *TP53* mutation statuses. Then, the UALCAN web resource was employed to investigate candidate gene expression levels among 1,097 BC samples of TCGA data based on individual cancer stages with the “Expression Link” of “TCGA analysis module”.

### 2.9 Immune infiltration analysis

The “gene module” of “Immune Association” in Tumor Immune Estimation Resource, version 2.0^40^ (TIMER2.0, http://timer.cistrome.org/) ^40^ was used to estimate the correlations between the expression levels of candidate genes and the infiltration of CD8^+^ T cells, macrophages, NK cells, and cancer‐associated fibroblasts (CAFs) among four major subtypes of BC using all algorithms provided, like EPIC, TIMER, CIBERSORT, CIBERSORT‐ABS, QUANTISEQ, XCELL, and MCPCOUNTER algorithm with Spearman's rank correlation coefficient and statistical significance: *p* < 0.05. Moreover, for CD8^+^ T cells and macrophages, we further employed the Breast Cancer Gene‐Expression Miner v4.7 tool to estimate the correlations between the expression of candidate genes and the biomarkers of these two immune cells, as well as six immune checkpoints (*PDCD1*, *CD274*, *CTLA4*, *TIGIT*, *LAG3*, and *BTLA*).

### 2.10 Promoter methylation, genetic alteration, and TF‐targeted genes analysis

Promoter methylation analysis was implemented using UALCAN web based on TCGA data containing 793 BC and 97 normal samples. The genetic alteration analysis of candidate genes was carried out in cBioPortal^41^ web (https://www.cbioportal.org/) based on 8196 BC samples. For TF candidate genes, we applied TRRUST web to predict the potential downstream‐targeted genes and visualized with Cytoscape software. Meanwhile, the functional annotation of them was investigated with Metascape.

### 2.11 BC sample collection, mRNA extraction, and reverse transcription polymerase chain reaction

Four BC samples (1 luminal A, 2 luminal B, and 1 HER2^+^ subtypes) and paired normal breast samples, as well as five samples of benign breast disease, were obtained from patients at Zhongnan Hospital of Wuhan University with informed consent. The Ethics Committee of Zhongnan Hospital of Wuhan University approved the use of these samples for total RNA isolation and reverse transcription polymerase chain reaction (RT‐PCR) analysis. The tissues were instantly frozen by liquid nitrogen and then stored at −80°C until used. Total RNA was isolated from the tissues and then converted to complementary DNA (cDNA) with the HiScript II Q RT SuperMix (Vazyme) according to the manufacturer's instruction. The real‐time RT‐PCR was implemented using the Universal SYBR Green Fast qPCR Mix in triplicates. The 2^−ΔΔCT^ method was used to normalize each gene with *GAPDH* as internal control. The primer sequences for RT‐PCR used in this study were designed and selected from Primer designing tool (http://www.ncbi.nlm.nih.gov/tools/primer‐blast/) and listed in Table S1.

### 2.12 Gene set enrichment analysis

Gene set enrichment analysis (GSEA)^42^ was used to assess pathway variations in these three datasets via dividing para‐cancerous and BC samples into high/low‐expression groups according to the median expression value of *FOS*, respectively, and with the cutoff: false discovery rate (FDR) *q* < 0.25.

## 3 RESULTS

### 3.1 Raw data quality control

Based on the box charts of NUSE, there was no aberrant chip in the GSE9574 dataset, 1 aberrant chip from para‐cancer tissues in GSE15852, and 13 aberrant chips in GSE42568, consisting of three normal breast tissue and 10 BC chips. After excluding the abnormal samples, the median standard error of all gene chips shown in the NUSE boxplots (Figure S1A–C) was close to 1 and no more than 1.05, indicating a good agreement among the rest samples. With RNA degradation plots, one chip from tumor paired normal tissues in GSE15852 was inconsistent with others and had a lower slope compared to the other chips. RNA degradation of the remaining gene chips was considered to be acceptable (Figure S1D–F). Therefore, we guaranteed the quality of raw data and made them more rigorous.

### 3.2 Raw data standardization and probe set annotation

Raw data were processed with the rma algorithm of R package affy and interpreted probe set expression to gene expression profile. As a result, a total of 12,413 genes were detected in GSE9574 and GSE15852 based on GPL96, while 20,460 genes in GSE42568 based on GPL570.

### 3.3 DEG identification

**FIGURE 1:**
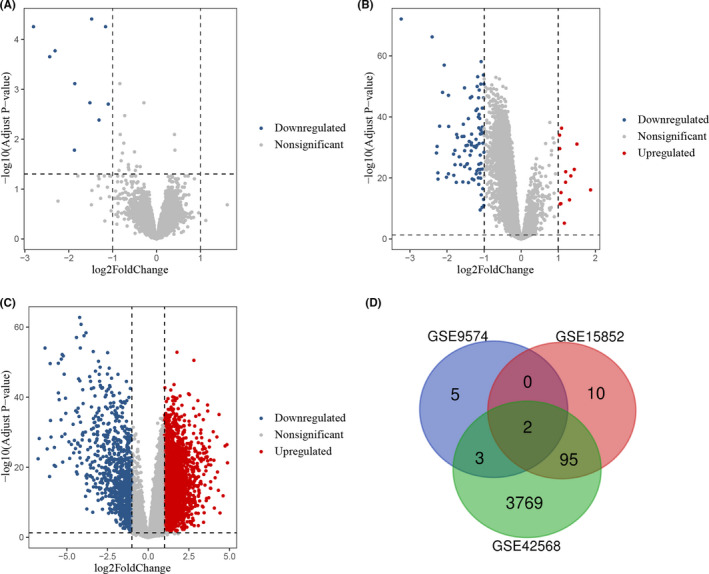
Differentially expressed gene (DEG) identification for each GEO dataset. (A–C) Volcano plots of all genes detected by probes in GSE9574, GSE15852, and GSE42568 datasets, according to the criterion: |log2FC| > 1 and adjusted *p* < 0.05. The red dots represent upregulated DEGs, the gray dots represent genes without significant difference between groups, and the blue dots represent downregulated DEGs. (D) The Venn diagram of DEGs identified from the three datasets

In GSE9574 dataset, only 10 DEGs were extracted, and all of them were downregulated in the para‐cancerous compared with breast normal tissues (Figure 1A). In GSE15852 dataset, 14 upregulated and 93 downregulated genes in BC compared with para‐cancerous tissues were identified (Figure 1B), while 2936 upregulated and 933 downregulated genes in BC compared with normal tissues were obtained in GSE42568 (Figure 1C). In addition, through further comparison among these DEGs, we found that two downregulated DEGs (*FOS* and *FOSB*) were overlapped within all the three datasets. In addition, there were other three common downregulated DEGs (*ZFP36*, *EGR1*, and *JUN*) between GSE9574 and GSE42568 datasets, and 95 shared DEGs between GSE15852 and GSE42568 datasets, of which 13 were upregulated and 82 downregulated (Figure 1D; Table 1).

**TABLE 1:** Overlapping differentially expressed genes in each dataset

Datasets	Upregulated genes	Downregulated genes
GSE9574&GSE15852&GSE42568		*FOS FOSB*
GSE9574&GSE42568		*ZFP36 EGR1 JUN*
GSE15852&GSE42568	*DSP GATA3 CD24 KRT18 BUB1B KRT19 EFNA1 SLC38A1 S100A14 SPP1 CD9 TFF3 EPCAM*	*ESYT1 DPT CHRDL1 PRKAR2B ITIH5 ECM2 ITGB1BP1 LEP SERPINF1 ECH1 RBP4 S100A4 PYGL EPB41L2 GHR GNAI1 GBE1 LGALS1 MAOA ALDH2 IGFBP6 PPAP2A PALMD AKAP12 CD36 ASS1 CAV1 EGFL6 ACSL1 PPARG PC PDZD2 ADIRF HBB GNG11 FHL1 SRPX COX7A1 C2CD2 FTL NEK7 PLSCR4 ABCA8 AOC3 ADH1B AKR1C1 PEMT MMD LPL PTPRM VIM FABP4 ACACB TNMD PPP1R1A ADCK3 PCK1 RGCC CIDEC RRAS HSD11B1 PFKFB3 AKR1C3 ITGA7 SPARC ECHDC3 F3 G0S2 PCOLCE2 ADAMTS5 RETSAT WASF3 GYG2 FOXO1 CDH5 CFD TMEM100 FAM13A EFEMP1 PLIN1 COPG2IT1 ADIPOQ*

### 3.4 GO terms and pathways enriched by DEGs

**FIGURE 2:**
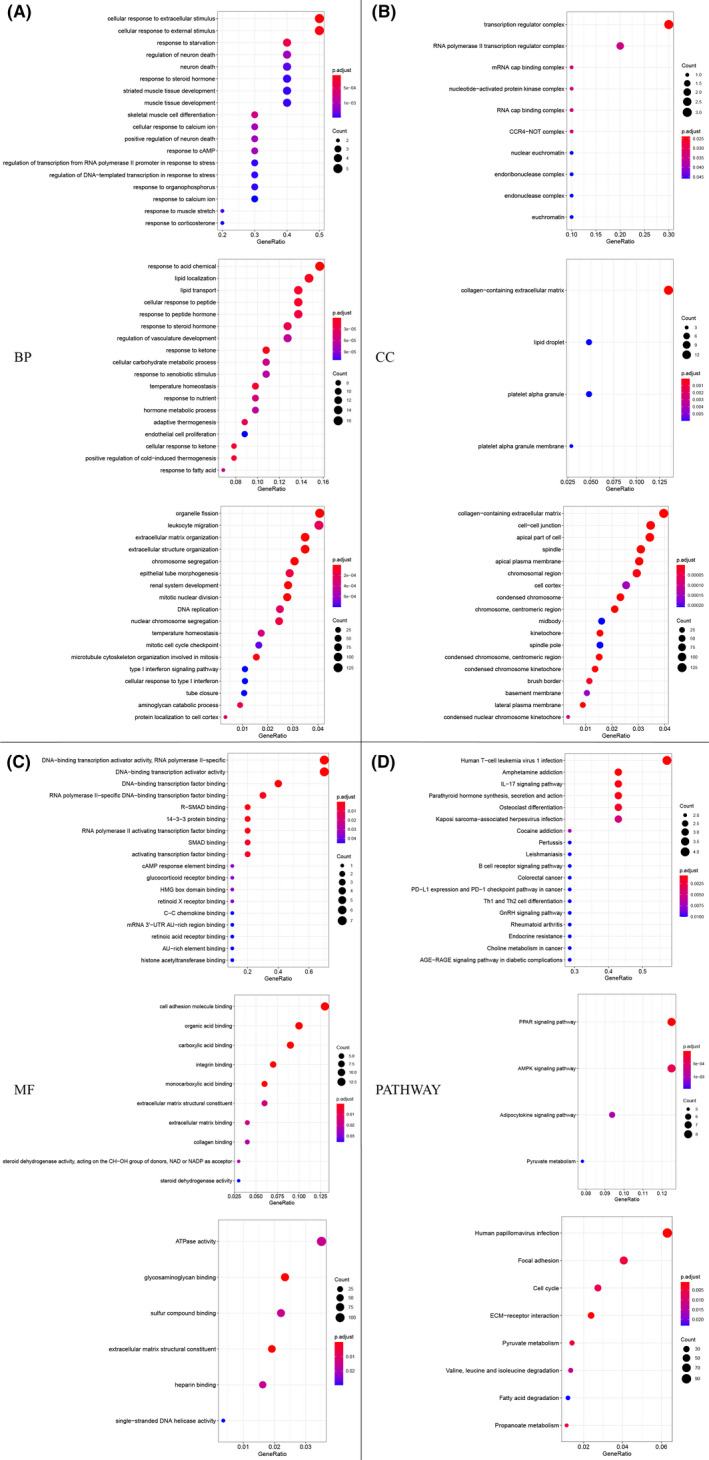
Gene Ontology (GO) function and Kyoto Encyclopedia of Genes and Genomes (KEGG) pathway enrichment analyses of DEGs. (A) The top 18 biological process terms of GO enriched by most differentially expressed genes (DEGs) in GSE9574, GSE15852, and GSE42568 datasets, respectively. (B) The major cellular components terms of GO enriched by DEGs in GSE9574, GSE15852, and GSE42568. (C) The main molecular functions of DEGs in GSE9574, GSE15852, and GSE42568. (D) The significantly enriched KEGG pathways of DEGs in GSE9574, GSE15852, and GSE42568. BP, biological processes; CC, cellular components; MF, molecular functions

To further explore the mechanism of the occurrence and development of BC, we conducted GO function and KEGG pathway annotation for the DEGs extracted from these three datasets. We discovered that 112 main biological processes (BP) were significantly affected in para‐cancerous compared to normal tissues with adjusted *p* < 0.05, of which the top three were cellular response to extracellular stimulus, cellular response to external stimulus, and response to starvation. Compared to para‐cancerous tissues, 175 main BP were affected in BC tissues, of which the top three were response to acid chemical, lipid localization, and lipid transport. Compared to normal tissues, 164 main BP were influenced in BC tissues, the top three of which were organelle fission, leukocyte migration, and extracellular matrix organization (Figure 2A). Most DEGs identified from GSE9574 or their corresponding proteins were elements of transcription regulator complex, RNA polymerase II transcription regulator complex, and mRNA cap‐binding complex (top three of 10 in total). While the majority of DEGs from GSE15852 were mainly constituents of collagen‐containing extracellular matrix, lipid droplet, platelet alpha granule, and platelet alpha granule membrane, and most DEGs from GSE42568 were main contributors of collagen‐containing extracellular matrix, cell–cell junction, and apical part of cell (Figure 2B). Meanwhile, these DEGs from these datasets also had diversely main molecular functions (Figure 2C).

With KEGG pathway analysis, we found 40 pathways significantly gathered by DEGs of GSE9574, the top three of which were human T‐cell leukemia virus 1 infection, amphetamine addition, and IL‐17 signaling pathway. The DEGs of GSE15852 were mainly gathered in the peroxisome proliferator‐activated receptors (PPAR) signaling pathway, AMPK signaling pathway, adipocytokine signaling pathway, and pyruvate metabolism pathway. The eight pathways significantly enriched by DEGs of GSE42568 were human papillomavirus infection, focal adhesion, cell cycle, ECM‐receptor interaction, pyruvate metabolism, valine, leucine and isoleucine degradation, fatty acid degradation, and propanoate metabolism (Figure 2D).

### 3.5 Network construction and module gene identification

**FIGURE 3:**
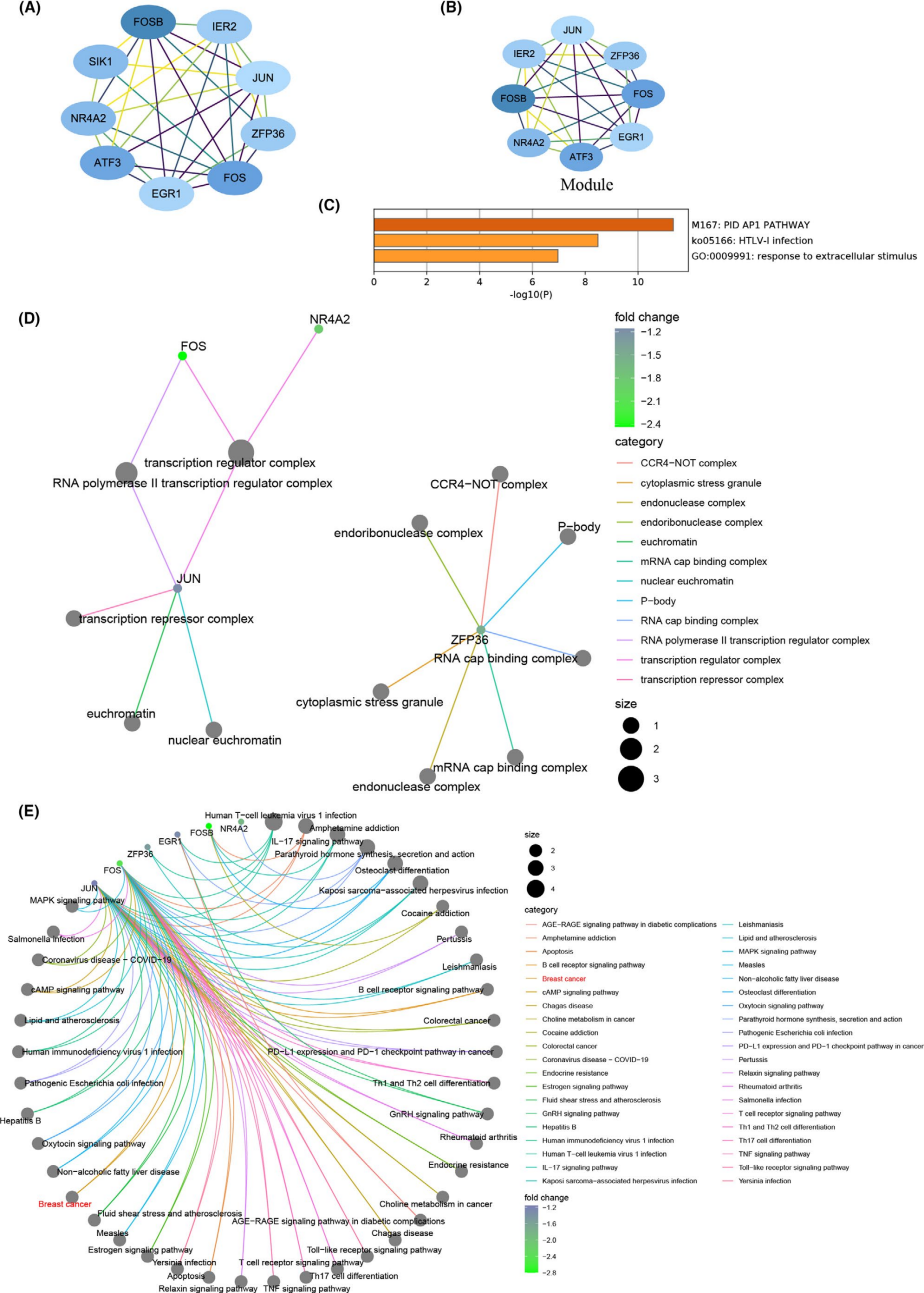
PPI network and module analysis of differentially expressed genes (DEGs) identified from GSE9574 dataset. (A) PPI network constructed by DEGs from GSE9574 dataset. Each node represents a gene. The blue nodes denote the downregulated genes in adjacent‐tumor tissues compared with normal tissues. The darker the nodes, the more differentially expressed the genes between groups. The degrees between them indicate their interactions. (B) A module identified from PPI network. (C) Multiple functions and pathways enriched by module genes. (D) cellular components (CC) terms of Gene Ontology enriched by module genes. (E) Kyoto Encyclopedia of Genes and Genomes pathways enriched by module genes

To estimate the relationships or interactions among products encoded by DEGs and further screen out candidate genes, we constructed PPI networks by DEGs identified from GSE9574 and GSE15852. A network containing nine nodes and 30 edges were created with DEGs of GSE9574 (Figure 3A), from which a module was recognized with MCODE scores = 7.429 including eight nodes (i.e., eight downregulated DEGs, *NR4A2*, *FOSB*, immediate early response 2 [*IER2*], *ZFP36*, *FOS*, *EGR1*, *ATF3*, and *JUN*) and 26 edges (Figure 3B). Furthermore, multiple function enrichment analysis was performed on module genes using Metascape resource, which mainly gathered in PID‐AP1 pathway, HTLV‐1 infection, and response to extracellular stimulus (Figure 3C; Table S2). In addition, FOS, NR4A2, and JUN are members of the transcription regulator complex. JUN also participates in transcription repression, and ZFP36 was reported to compose mRNA cap‐binding complex (Figure 3D). JUN and FOS were involved in lots of pathways, including MAPK signaling pathway, PD‐L1 expression and PD‐1 checkpoint pathway in BC, colorectal cancer, and even coronavirus disease––COVID‐19 pathway (Figure 3E).

**FIGURE 4:**
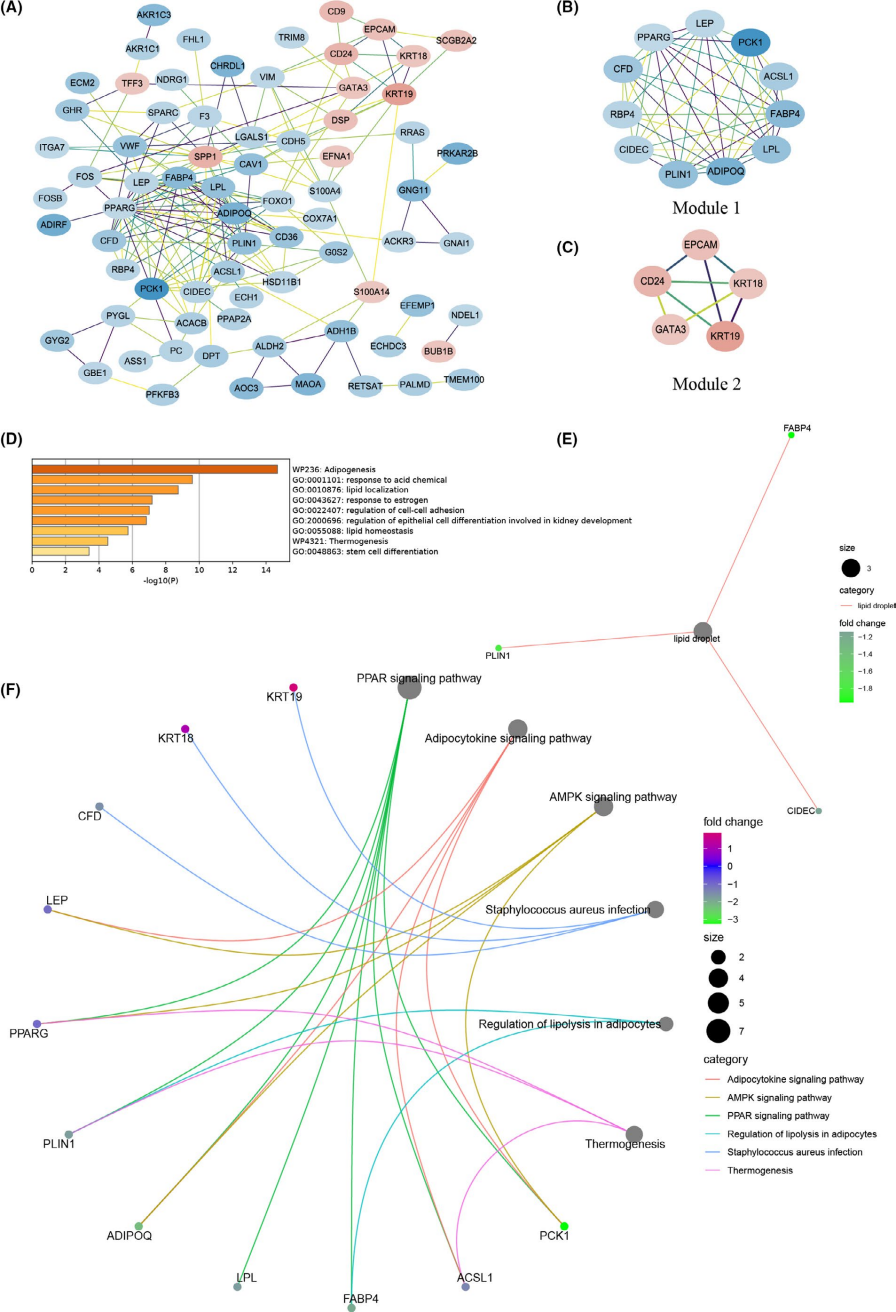
PPI network and module analysis of differentially expressed genes (DEGs) identified from GSE15852 dataset. (A) PPI network constructed by DEGs from GSE15852 dataset. Each node represents a gene. The blue nodes represent the downregulated genes, and the red nodes represent the upregulated genes in BC tissues compared with adjacent‐tumor tissues. The darker the nodes, the more differentially expressed the genes between groups. The degrees between them indicate their interactions. (B, C) Two modules identified from PPI network. (D) Multiple functions and pathways enriched by module genes. (E) Cellular components terms of Gene Ontology enriched by module genes. (F) Kyoto Encyclopedia of Genes and Genomes pathways enriched by module genes

From the network structured by 74 nodes and 194 edges with DEGs of the GSE15852 dataset (Figure 4A), three modules were identified, of which the first two were selected to further analysis with MCODE scores = 9.400 and 4.000. One was formed by 11 nodes (i.e., 11 downregulated DEGs, *ACSL1*, *PCK1*, *CIDEC*, *RBP4*, *CFD*, *LEP*, *LPL*, *FABP4*, *PLIN1*, *ADIPOQ*, and *PPARG*) and 47 edges (Figure 4B). The other was composed of five nodes and eight edges, and all DEGs (*KRT18*, *CD24*, *KRT19*, *EPCAM*, and *GATA3*) were upregulated (Figure 4C). These module genes were mainly enriched in adipogenesis pathway, response to acid chemical term of GO, lipid localization of GO (Figure 4D; Table S3). Moreover, we also found that PLIN1, FABP4, and CIDEC were located in lipid droplet (Figure 4E) and PPARG, PLIN1, ADIPOQ, LPL, FABP4, ACSL1, and PCK1 were involved in the PPAR signaling pathway.

**FIGURE 5:**
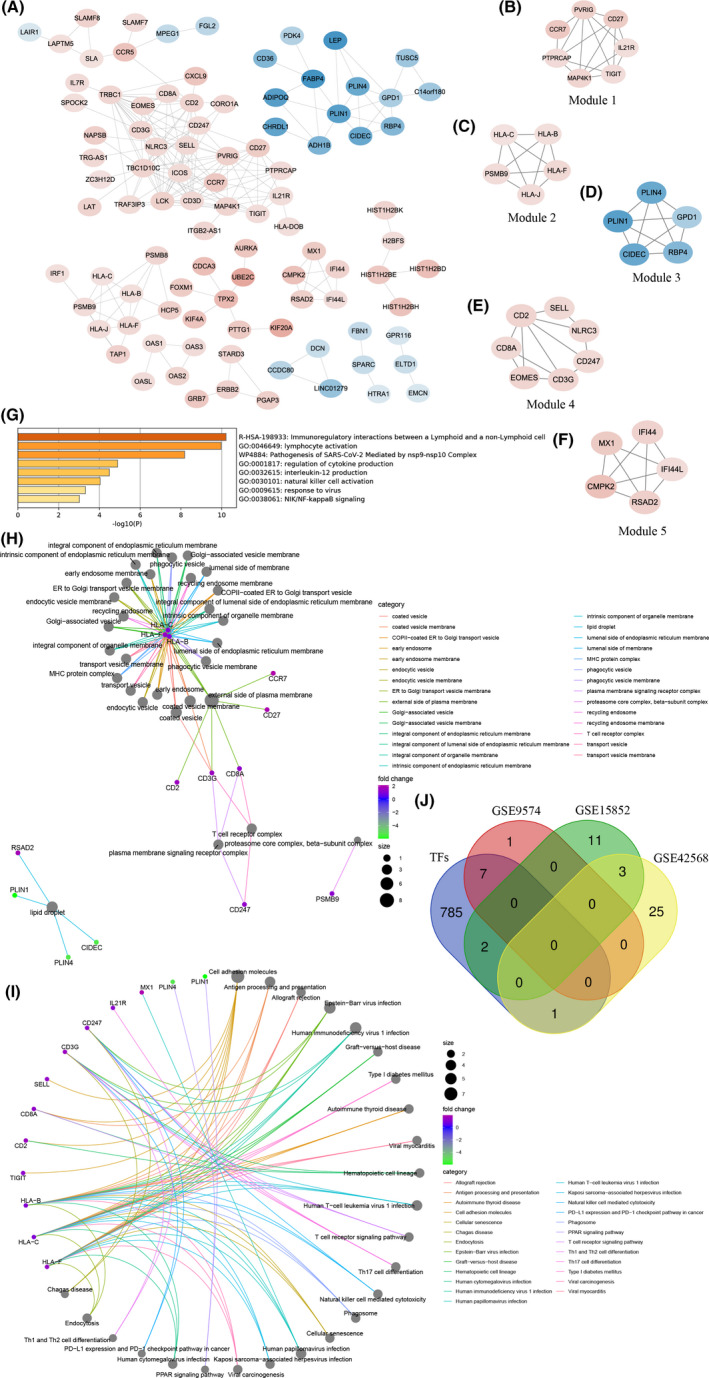
Gene co‐expression network and module analysis of differentially expressed genes (DEGs) identified from GSE42568 dataset and transcription factor screening. (A) Gene co‐expression network formed by DEGs from GSE42568 dataset. Each node represents a gene. The blue nodes represent the downregulated genes, and the red nodes represent the upregulated genes in BC tissues compared with adjacent‐tumor tissues. The darker the nodes, the more differentially expressed the genes between groups. The degrees between them indicate their co‐expressive relationships. (B‐F) Five modules identified from the co‐expression network. (G) Multiple functions and pathways enriched by module genes. (H) Cellular components terms of Gene Ontology enriched by module genes. (I) Kyoto Encyclopedia of Genes and Genomes pathways enriched by module genes. (J) Venn diagram of transcription factors download from TRRUST database and module genes extracted from GSE9574, GSE15852, and GSE42568 datasets

As for the DEGs of the GSE42568 dataset, the number of them was too large to create a PPI network using STRING. Therefore, we performed WGCNA to generate a gene co‐expression network with power = 6, minimum size = 4, and threshold = 0.4 (Figure S2), and subsequently to mine module genes via applying plug‐in MCODE in Cytoscape. In consequence, five modules including 29 genes were sorted out with MCODE scores = 5.333, 5.000, 5.000, 4.500, and 4.000, respectively (Figure 5A–F). Involved genes were mainly aggregated in “immunoregulatory interactions between a Lymphoid and a non‐Lymphoid cell” of “Reactome Gene Sets”, “lymphocyte activation” of “GO BP”, and “pathogenesis of SARS‐CoV‐2 mediated by nsp9‐nsp10 complex” of “WikiPathways” (Figure 5G; Table S4). In addition, HLA‐B, HLA‐C, and HLA‐F contributed to multiple cellular components, like MHC protein complex, ER to Golgi transport vesicle membrane, early endosome, and others. Meanwhile, CCR7, CD27, CD2, CD3G, and CD8A were constituents of external side of plasma membrane, as well as HLA‐B, HLA‐C, and HLA‐F (Figure 5H). Moreover, CD247, CD3G, HLA‐B, HLA‐C, and HLA‐F took part in various pathways, including Th1 and Th2 cell differentiation, PD‐L1 expression and PD‐1 checkpoint pathway in cancer, Th17 cell differentiation, antigen processing presentation, allograft rejection, and cell adhesion molecular (Figure 5I).

Furthermore, through TRRUST database we found that seven genes (*JUN*, *FOS*, *FOSB*, *ATF3*, *EGR1*, *NR4A2*, and *ZFP36*) identified from GSE9574, two genes (*GATA3* and *PPARG*) from GSE15852, and one gene *EOMES* from GSE42568 were TFs (Figure 5J).

### 3.6 Module gene expression validation and prognostic values for BC patients

A total of 50 unique module genes in PPI and gene co‐expression networks were identified from three GEO datasets covering breast tissues in different statuses. To further confirm our findings, UALCAN web was applied to investigate the distinctions of their transcriptional levels between BC and normal samples based on TCGA. The expressions of all module genes were significantly discrepant by comparing normal samples with tumor, including three genes (*ATF3*, *IER2*, and *NNR4A2*) downregulated in para‐cancerous compared to normal tissues, which were also low expressed in BC compared to normal samples. Whereas, we noted that five genes (*HLA‐J*, *NLRC3*, *CD247*, *CD3G*, and *CD8A*) turned to be downregulated in BC compared with normal tissues, which were opposite to the result of GSE42568 of the GEO database (Table S5). Thus, they were excluded from the following analysis.

**FIGURE 6:**
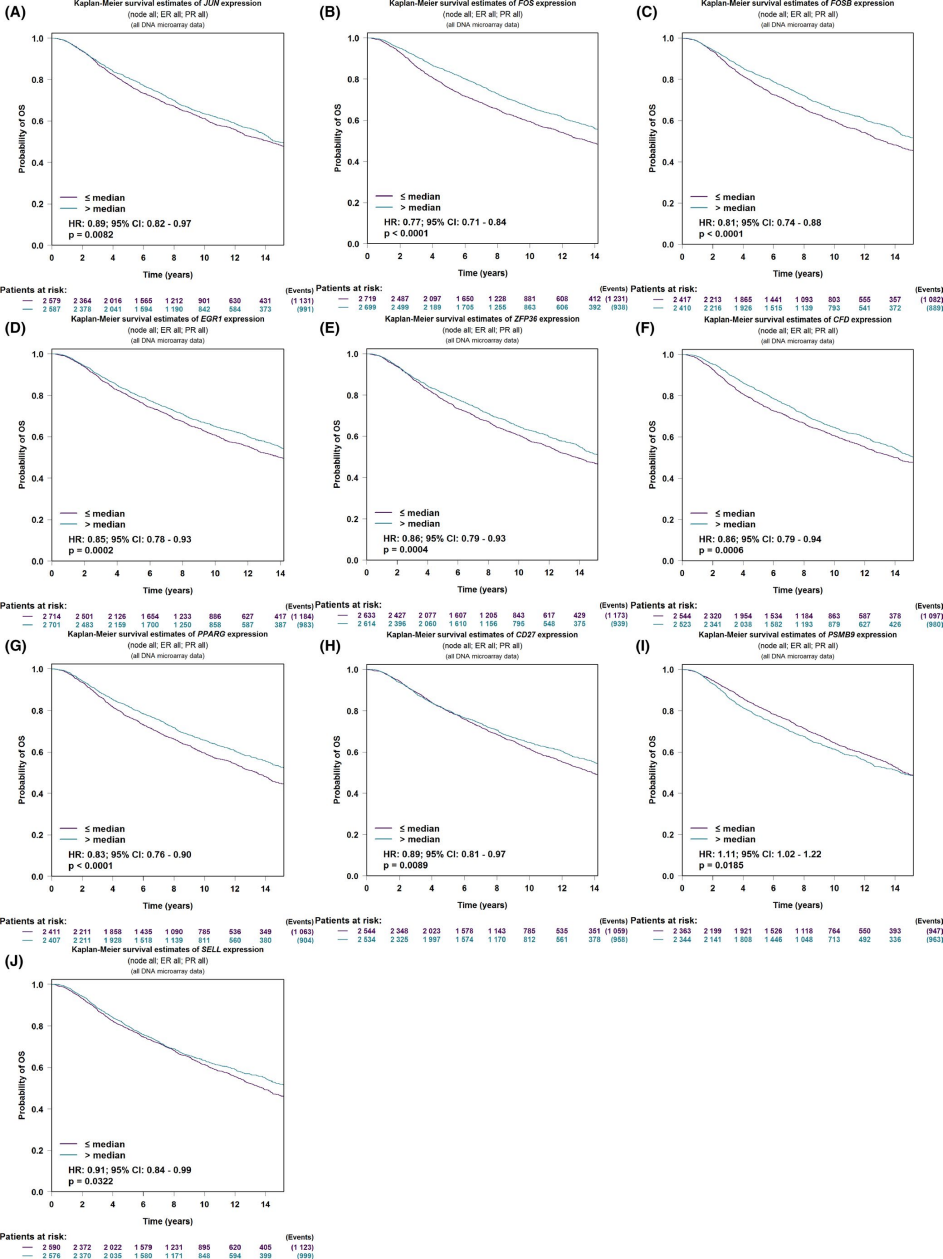
Associations of candidate genes with the overall survival (OS) in breast cancer (BC) patients. (A–E) Five candidate genes JUN, *FOS*, *FOSB*, *EGR1*, and *ZFP36* from the GSE9574 dataset significantly associated with the OS in BC patients. (E–J) Five candidate genes *CFD*, *PPARG*, *CD27*, *PSMB9*, and *SELL* were significantly associated with the OS evaluated by multivariate Cox survival analysis in BC patients

Subsequently, the OS, DMFS, and RFS were analyzed using the bc‐GenExMiner v4.7 resource based on all DNA microarray data. For module genes from GSE9574, five genes *JUN*, *FOS*, *FOSB*, *EGR1*, and *ZFP36* were positively associated with OS and DFS, among which four genes (*FOS*, *FOSB*, *EGR1*, and *ZFP36*) were also positively associated with DMFS with statistical significance *p* < 0.05. For module genes from GSE15852, 13 genes were associated with OS, of which the high expressions of *CFD*, *LEP*, *LPL*, *FABP4*, *PLIN1*, *ADIPOQ*, *PPARG*, and *GATA3* predicted better prognosis, while *ACSL1*, *CD24*, *KRT18*, *KRT19*, and *EPCAM* were reverse. Among them, eight and 10 genes were significantly correlated with DMFS and DFS, respectively. For module genes from GSE42568, except for the overlapping gene *PLIN1*, 11 genes were related to OS, of which *PVRIG*, *CD27*, *GPD1*, *PLIN4*, and *SELL* were positively associated with OS, while *PSMB9*, *CMPK2*, *RSAD2*, *MX1*, *IFI44*, and *IFI44L* were reverse. Among them, nine and 11 genes were also significantly correlated with DMFS and DFS, respectively. (Figure 6; Table S5).

Moreover, we further predicted the prognostic values of these genes associated with OS in BC patients with different stages, ages, molecular subtypes, ER/PR/HER2 status, TNM status, and *TP53* mutation status, in addition to their expression levels using Cox Proportional‐Hazards model based on TCGA cohorts. As a result, a total of 789 BC patients were enrolled, and the clinical characteristics and *TP53* status are listed in Table S6. According to the univariate Cox regression analysis, we found that stage, age, tumor status, node status, metastasis status, and the expression levels of *CD24* were significantly negatively correlated to OS as risk factors, while the expression levels of *JUN*, *FOS*, *FOSB*, *ZFP36*, *CFD*, *LEP*, *PLIN1*, *ADIPOQ*, *PPARG*, *PVRIG*, *CD27*, *PSMB9*, *GPD1*, *PLIN4*, and *SELL* were significantly positively correlated to OS as protective factors (Table S7). Then, according to multivariate analysis, only *CFD* (HR = 0.84, *p* = 0.0327), *PPARG* (HR = 0.80, *p* = 0.0478), *CD27* (HR = 0.75, *p* = 0.0001), *PSMB9* (HR = 0.73, *p* = 0.00299), and *SELL* (HR = 0.77, *p* = 0.0062) were still significantly positively associated with OS as potentially independent prognostic factors in BC patients (Table S8).

### 3.7 Expression pattern of candidate genes based on clinical features and association with immune microenvironment in BC

**FIGURE 7:**
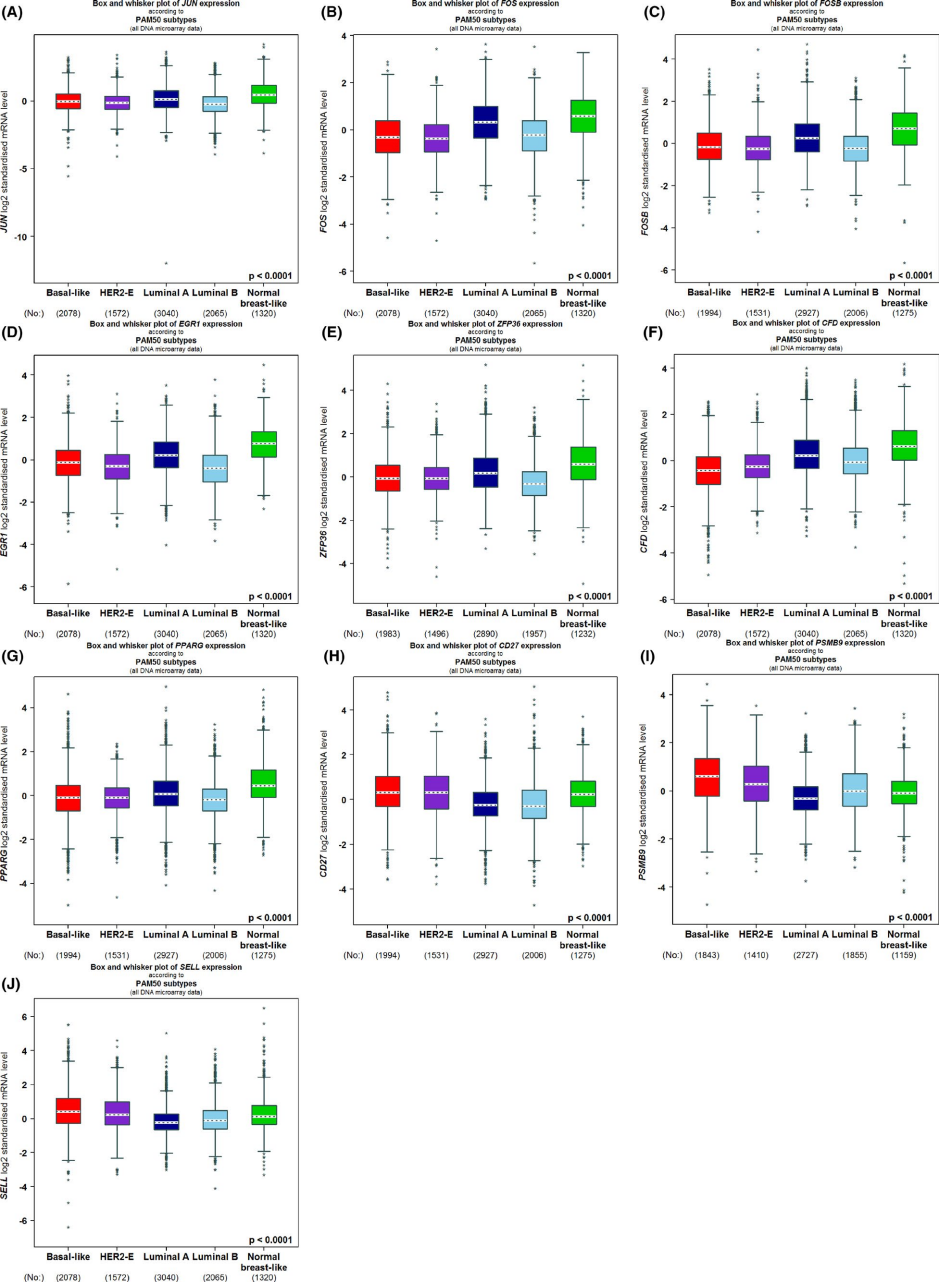
Expression levels of candidate genes based on major subclasses of breast cancer (BC). (A–E) Five candidate genes JUN, *FOS*, *FOSB*, *EGR1*, and *ZFP36* from GSE9574 dataset (F–G) Two candidate genes *CFD* and *PPARG* from GSE15852 dataset (H–J) Three candidate genes *CD27*, *PSMB9*, and *SELL* from GSE42568 dataset

Through expression validation and survival analysis, 10 candidate genes (*JUN*, *FOS*, *FOSB*, *EGR1*, *ZFFP36*, *PPARG*, *CFD*, *CD27*, *PSMB9*, and *SELL*) were determined. To dissect their variations and roles in BC, we further explored their expression levels based on clinical features, and whether their aberrations were correlated with BC immune microenvironment (CD8^+^ T cell, macrophage, NK cell, and CAF) in four main subtypes of BC. As a result, seven candidate genes (*JUN*, *FOS*, *FOSB*, *EGR1*, *ZFP36*, *CFD*, and *PPARG*) were significantly upregulated in luminal A type of BC and normal breast‐like samples compared with luminal B, HER2^+^, and TNBC, while *CD27*, *PSMB9*, and *SELL* were opposite (Figure 7), as well as in *TP53*‐normal relative to *TP53*‐mutant group (Figure S3). The expressions levels of *FOS*, *FOSB*, *EGR1*, *JUN*, *ZFP36*, *PPARG*, and *CFD* were lower as the stage got worse, whereas the expression of *PSMB9* was higher (Figure S4). Meanwhile, *JUN*, *FOS*, *EGR1*, *ZFP36*, *CFD*, and *PPARG* were lower expressed in patients aged 21–40 compared to 40–70 and 70–97, while *CD27*, *PSMB9*, and *SELL* were higher expressed (Figure S5). In addition, *FOS* and *FOSB* were downregulated when lymph node metastasis was present, while *CD27* and *SELL* were upregulated (Figure S6).

**FIGURE 8:**
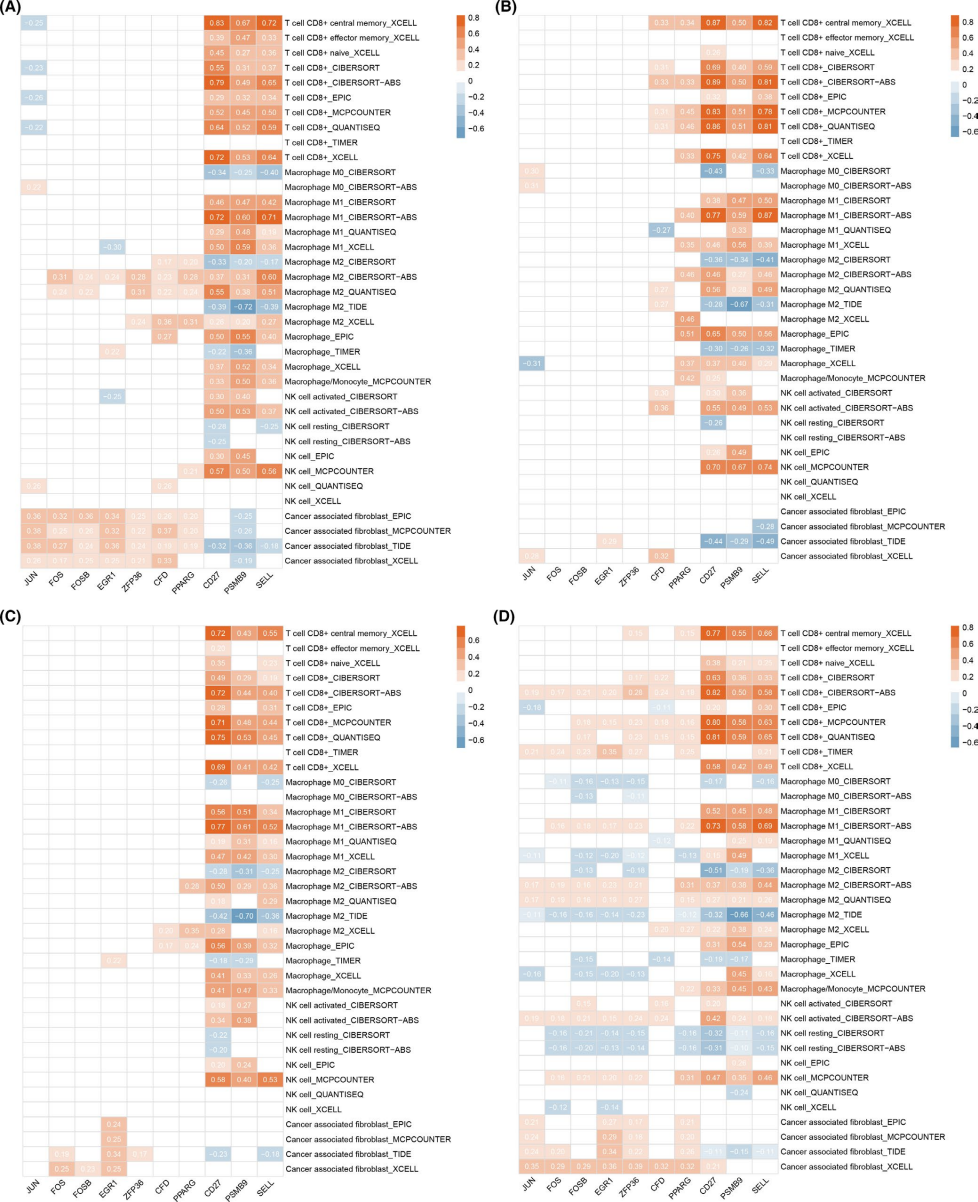
Correlations between candidate gene expression and immune cell infiltration (CD8^+^ T cell, macrophage, natural killer cell, and cancer‐associated fibroblast) in four major types of breast cancer (BC). (A) In basal BC. (B) In HER2^+^ BC. (C) In luminal A type of BC. (D) In luminal B type of BC. The color of the rectangle indicates the corresponding degree of correlation. The number in the rectangle represents the corresponding Spearman correlation coefficient and those with *p* < 0.05 are presented

Next, we estimated the correlations of candidate gene expression levels with infiltration levels of four immune cells (CD8^+^ T cell, macrophage, NK cell, and CAF) in four subtypes of BC, respectively. Multiple algorithm results displayed that *JUN* was negatively correlated with CD8^+^ T cell infiltration in basal BC, while positively with CAF infiltration in basal and luminal A type of BC (Figure 8A,D). *FOS*, *FOSB*, *EGR1*, *ZFP36*, *CFD*, and *PPARG* were positively correlated with M2 macrophage and CAF infiltration in basal BC, while they were positively correlated with CD8^+^ T cells, activated NK cells, and CAFs, and negatively correlated with resting NK cells in luminal A type of BC (Figure 8A,D). Of interest, the correlations of *CD27*, *PSMB9*, and *SELL* expression with immune cell infiltration were almost the same among four subtypes of BC (Figure 8). Additionally, we noted that *CFD* and *PPARG* were positively correlated with CD8^+^ T cells, macrophages, activated NK cells, and CAFs in HER2^+^ type of BC, but only with macrophages in luminal B type of BC (Figure 8B,C). *FOS*, *FOSB*, *EGR1*, and *ZFP36* were positively correlated with CAFs in luminal B type of BC (Figure 8C). Furthermore, *CD27*, *PSMB9*, and *SELL* were positively correlated with the biomarkers of CD8^+^ T cells and macrophages, as well as immune checkpoints, such as *CTLA4*, *TIGIT*, and *BTLA* (Figure S7).

### 3.8 Promoter methylation, genetic alteration, and downstream‐targeted genes of candidate genes

Then, we explored the mechanism of candidate gene expression changes from DNA promoter methylation status and genetic alteration. The promoter methylation levels of three genes *PPARG*, *CFD*, and *SELL* were significantly higher in BC than normal samples (Figure S8A–C), and the main genetic alterations of these candidate genes in BC were amplification and deep deletion. Nine genes existed amplification with the highest alteration frequency, especially *SELL* with almost 14% amplification, while *CFD* existed more deep deletion.

**FIGURE 9:**
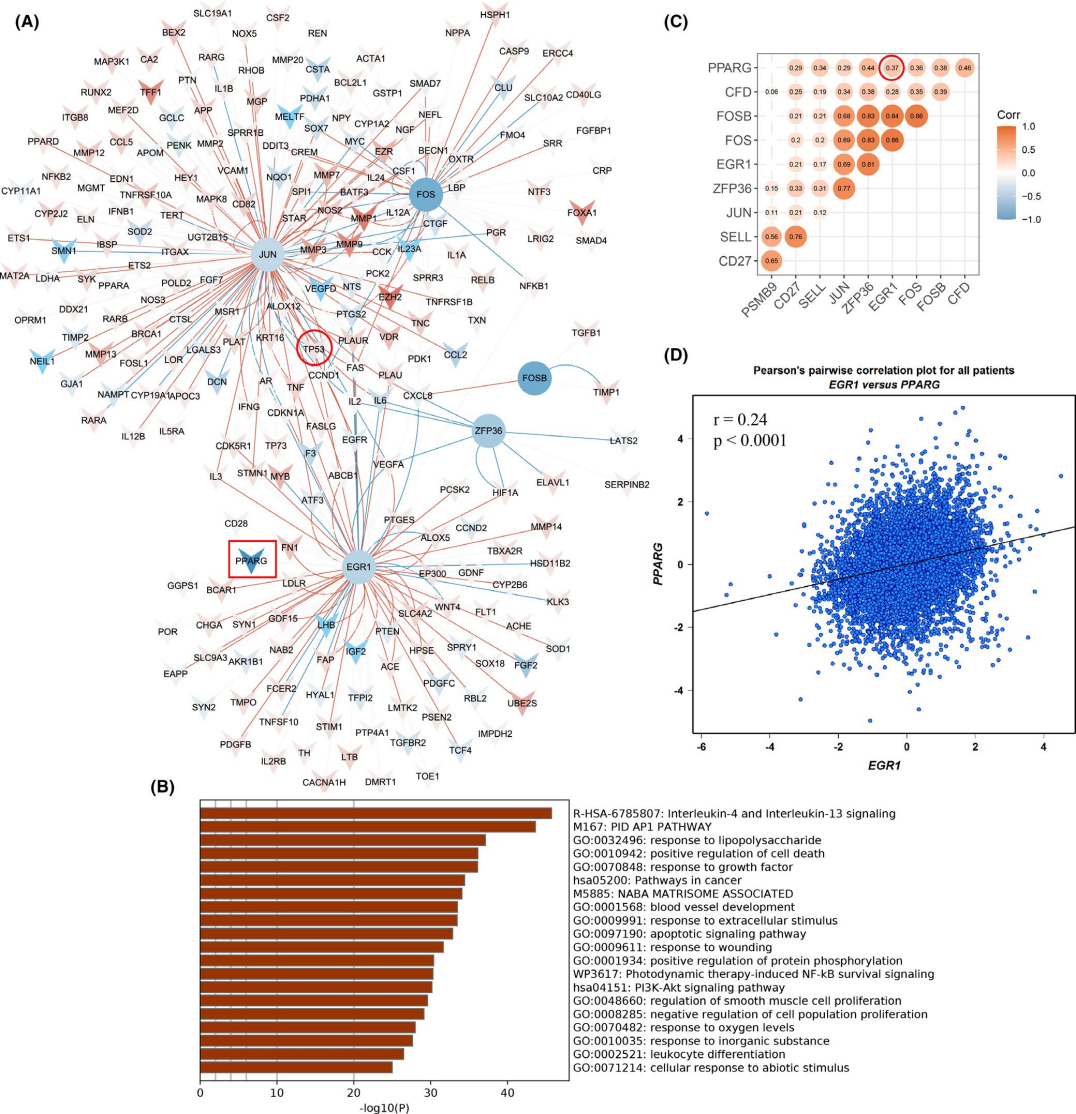
Downstream‐targeted genes of transcription factors, function enrichment, and correlation analysis. (A) Downstream‐targeted genes of *JUN*, *FOS*, *FOSB*, *EGR1*, and *ZFP36*. The circles represent transcription factors and the V shapes represent targeted genes, those with red indicate the corresponding genes are upregulated in BC compared with normal samples, while those with blue indicate the corresponding genes are downregulated. The red connectors present activation, while the blue connectors present suppression. (B) Main functions enriched by downstream‐targeted genes of *JUN*, *FOS*, *FOSB*, *EGR1*, and *ZFP36*. (C) Expression correlations of candidate genes. The number in the circle represents the corresponding Pearson correlation coefficient and those with *p* < 0.05 are presented. (D) Expression correlation of *EGR1* with *PPARG*

After TF identification, we noted that seven out of eight module genes from GSE9574 were TFs, and through survival analysis, five of them (*JUN*, *FOS*, *FOSB*, *EGR1*, and *ZFP36*) were associated with the OS of BC patients. Therefore, we explored their downstream‐targeted genes (Figure 9A) and investigated functions and pathways enriched by these targeted genes (Figure 9B), including “positive regulation of cell death”, “pathways in cancer”, and “PI3K‐Akt signaling pathway”. Among these targeted genes, we noted *TP53* regulated by *JUN*, and *PPARG* regulated *by EGR1* were included (Figure 9A). Thereby, we further estimated expression correlations of candidate genes (Figure 9C) and found that the Pearson correlation coefficient between *EGR1* and *PPARG* was 0.37 with *p* < 0.05 based on the TCGA cohort. Therefore, we further validated their correlation using the bc‐GenExMiner v4.7 resource with *r* = 0.24 and *p* < 0.0001 (Figure 9D).

### 3.9 Expression validation of candidate genes among BC samples by RT‐PCR

To validate our findings in this study, we collected four BC samples (one luminal A, two luminal B, and one HER2^+^ subtype) and four paired normal breast samples, and five samples of benign breast disease. *JUN*, *FOS*, *FOSB*, *EGR1*, *ZFP36*, and *PPARG* were detectable in luminal A, luminal B, and HER2^+^ BC patients. All of them were downregulated in HER2+ and at least one luminal B patients by comparing cancer tissue to the corresponding normal tissue, except that *FOSB* was only downregulated in HER2^+^, and that *PPARG* was downregulated in luminal A and one luminal B patients (Figure S9A–F without *p*‐value). For *CFD*, *CD27*, *PSMB9*, and *SELL*, whose expression data were only available in HER2^+^ BC, and the results were consistent with those predicted by microarray data (Figure S9G without *p*‐value). Next, we compared expression levels of candidate genes in BC and non‐BC tissues obtained from patients with benign breast disease. All downregulated genes were lower expressed in cancer tissues from the HER2^+^ BC patient compared to non‐BC tissues, and all upregulated genes were obviously higher in cancer tissues from one luminal B BC patient (Figure S10).

### 3.10 Pathway variations correlated with the expression of *FOS*

**FIGURE 10:**
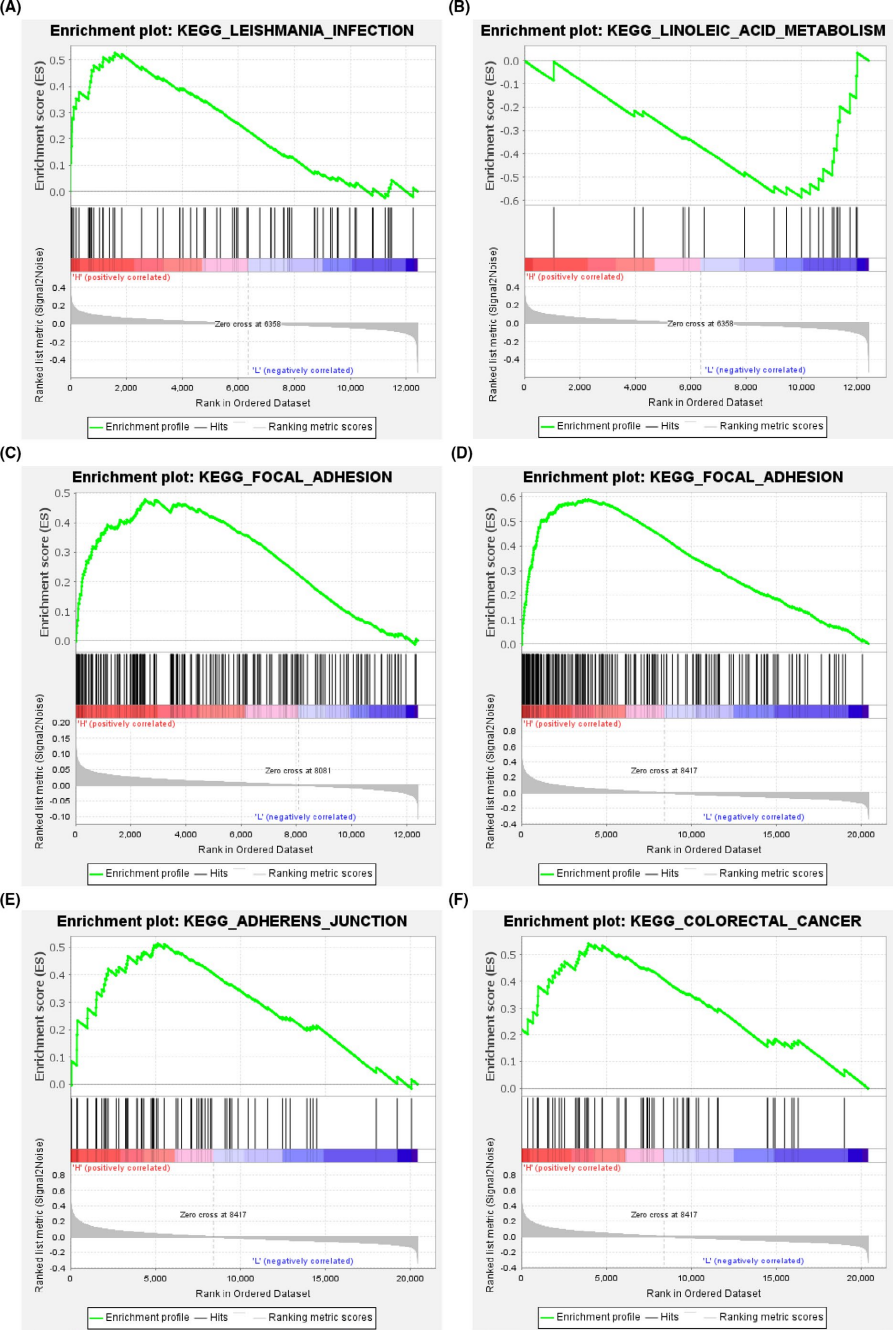
Gene set enrichment analysis based on the expression levels of *FOS* and the gene sets of KEGG pathways. (A) The top pathway positively correlated with the expression levels of *FOS* according to NES and false discovery rate (FDR) *q* < 0.25 based on the para‐cancerous samples in GSE9574 dataset. (B) The pathway negatively correlated with the expression levels of *FOS* according to FDR *q* < 0.25 based on the para‐cancerous samples in GSE9574 dataset. (C) The pathway positively correlated with the expression levels of *FOS* according to NES and FDR *q* < 0.25 based on the BC samples in GSE15852 dataset. (D–F) The top three pathways positively correlated with the expression levels of *FOS* according to NES and FDR *q* < 0.25 based on the BC samples in GSE42568 dataset

*FOS* was identified as a DEG in all the three datasets and positively associated with the OS, DMFS, and PFS in BC patients, as well as immune cell infiltration. *FOS* might play vital roles in neoplasia and progression in BC as a TF and immune‐associated gene. Thereby, we performed GSEA based on the expression values of *FOS* and discovered that nine pathways (containing “Leishmania infection”, “Fc gamma r mediated phagocytosis”, and “Antigen processing and presentation”) were positively correlated with the expression of *FOS* and one pathway (“Linoleic acid metabolism”) was negatively correlated in para‐cancerous samples of GSE9574 with the cutoff: FDR *q* < 0.25 (Figure 10A,B; Table S9). Diversely, in BC samples of GSE15852, two pathways (“Focal adhesion” and “Retinol metabolism”) were positively correlated with the expression of *FOS* (Figure 10C; Table S9), while in BC samples obtained from GSE42568, 14 pathways were positively correlated with the expression of *FOS*, of which the top three were “Focal adhesion”, “Adherens junction”, and “Colorectal cancer” according to normalized enrichment score (Figure 10D–F; Table S9).

## 4 DISCUSSION

BC is the most common carcinoma in women worldwide with the leading cause of cancer death.^1^ With the quick development of medical care, the mortality of BC has sharply decreased and approximately 70%–80% BC can be curable in recent years.^4^ However, a complete cure for BC patients is not yet possible with currently available therapies due to the ambiguous mechanism of tumorigenesis and tumor progression in BC. Consequently, it is necessary and a trend to dissect mechanisms of tumorigenesis and progression of BC more explicitly at the level of genes. In the present work, we focused on the transcriptional aberrations from normal tissues to BC.

First, we revealed different gene expression profiles among normal, para‐cancerous, and BC tissues, and initially identified 10 downregulated genes in para‐cancerous compared with normal samples, 14 upregulated and 93 downregulated genes in BC compared with para‐cancerous samples, and 2936 upregulated and 933 downregulated genes in BC compared with normal samples. Since para‐cancerous tissue is the intermediate stage from normal to BC. If the para‐cancerous tissue is not removed, cancer definitely will return.^43^ Thus, the disturbance in para‐cancerous tissue could help us unmask tumor formation. Following the thought, we further implemented function and pathway annotation analysis for these genes and found they participated in various BP, located in different cellular components, possessed diverse molecular functions, and were involved in different pathways, suggesting that in the process of tumor development, different stages had different main functional changes caused by these DEGs. Thereby, we next constructed PPI and gene co‐expression networks to show interrelationships among these DEGs, and further identify key genes (module genes). As a result, for the GSE9574 dataset, eight module genes were stood out and mainly involved in the AP1 pathway, HTLV‐1 infection, and response to extracellular stimulus. For GSE15852, 16 module genes were screened out and mainly involved in adipogenesis, response to acid chemical, regulation of cell–cell adhesion, etc. For GSE42568, 29 module genes were identified and mainly involved in immunoregulatory interactions between a lymphoid and a non‐lymphoid cell, NK cell activation, and NF‐kappa B signaling. These module genes might play crucial roles during tumorigenesis and progression of BC, through biological function and pathway perturbances caused by them.

TFs regulate gene expression and their regulatory networks are dynamic.^44^ In the study combined with the existed database of TFs, we found that seven module genes (*JUN*, *FOS*, *FOSB*, *ATF3*, *EGR1*, *NR4A2*, and *ZFP36*) identified from GSE9574 were TFs, while only two module genes (*GATA3* and *PPARG*) from GSE15852 and one module gene *EOMES* from GSE42568 were TFs, indicating that *JUN*, *FOS*, *FOSB*, *ATF3*, *EGR1*, *NR4A2*, and *ZFP36* might be responsible for tumor initiation even early before pathological diagnosis, and existed tumors might first control the expression of TFs and then influence the expression of their downstream target genes to create a suitable environment for survival and expansion.

Subsequently, with validations of the expression levels of these module genes and survival analysis, we further screened out 10 candidate genes for the following analysis. Five candidate genes (*JUN*, *FOS*, *FOSB*, *EGR1*, and *ZFP36*) were differentially expressed in para‐cancerous compared with normal tissues, and significantly associated with the OS of BC patients. The other five candidate genes (*CFD*, *PPARG*, *CD27*, *PSMB9*, and *SELL*) could be independent prognostic factors in BC. Among them, seven genes (*JUN*, *FOS*, *FOSB*, *EGR1*, *ZFP36*, *CFD*, and *PPARG*) were downregulated in BC compared to normal tissues, and in aggressive BC (basal, HER2^+^, and luminal B), *TP53* mutation group, younger patients, higher stages, and lymph node metastasis, while *CD27*, *PSMB9*, and *SELL* were upregulated, suggesting that these candidate genes could be indicators of aggressive types of BC, like HER2^+^ and TNBC, and might be responsible for the characteristics of invasive BC.

Prior studies noted that the immune system modulated the development and progression of BC, and in turn, cancer influenced the immune microenvironment.^45^ Previous studies discovered that immunity at early stages of cancer showed anticancer profiles, while as the tumor progressed, it could transfer to the tumor‐protective profile.^46^ Moreover, immune cell infiltrations, especially lymphocytes, were reported to be positively correlated with patients' outcomes at early stages of HER2+ BC and TNBC.^4^, ^47^, ^48^ On the contrast, the infiltration of CAFs and M2 macrophages may contribute to cancer progression.^49^, ^50^ The present study discovered that the expression levels of candidate genes were correlated with the infiltration of immune cells (CD8^+^ T cell, macrophage, NK cell, and CAF) in four subtypes of BC, especially in luminal A type of BC. We noted that correlations of three upregulated genes (*CD27*, *PSMB9*, and *SELL*) with immune cell infiltrations were almost the same in four subtypes of BC, while seven downregulated genes were not. Moreover, *CD27*, *PSMB9*, and *SELL* were also highly correlated with biomarkers of CD8^+^ T cells and macrophages, as well as immune checkpoints. These findings suggested that these candidate genes might be involved in tumor immuno‐editing and promisingly therapeutic targets for advanced BC.

Abnormal gene expression is regulated by genetic and epigenetic mechanisms in cancer.^51^, ^52^ Here, we further estimated promoter methylation and genetic alteration of candidate genes and found that the promoters of *PPARG*, *CFD*, and *SELL* were hypermethylated, while almost all of them existed amplification with highest frequency except for *CFD* with the high deep deletion. These results might partly explain the expression aberrations of candidate genes. Moreover, *JUN*, *FOS*, *FOSB*, *EGR1*, and *ZFP36* were differentially expressed in para‐cancerous compared with normal tissue and associated with OS of BC patients, which may participate in BC tumorigenesis. As all of them were TFs, we further explored downstream‐ targeted genes of them and found that *JUN*‐regulated *TP53* expression o, while *EGR1*‐regulated *PPARG* expression. These targeted genes were mainly involved in “positive regulation of cell death”, “pathways in cancer”, and “PI3K‐Akt signaling pathway”. These findings further revealed that these genes might be important for us to understand BC initiation. Moreover, we observed that the expression of *FOS* (one of the candidate genes and differentially expressed among breast tissues of three distinct statuses) was associated with different pathway variations in para‐cancerous and BC tissues through GSEA, indicating its various functions in BC.

In addition, for gene perturbations in para‐cancerous tissues, the original study identified 105 DEGs in histologically normal tissues near breast tumors compared to the normal tissues using Cyber‐*T*‐test and concluded that the characteristics of para‐cancerous tissues were more like BC tissues.^19^ Another study elucidated that expression profiles varied with distance from the tumor in eight BC patients.^53^ In our study, using R package limma with statistical significance: adjusted *p* < 0.05 and |log2 fold change| > 1, 10 DEGs were identified, and all of them were downregulated in tumor adjacent compared with the normal tissues, of which eight DEGs (*JUN*, *FOSB*, *FOS*, *ATF3*, *EGR1*, *IER2*, *ZFP36*, and *NR4A2*) were overlapped with the original study mentioned above.^19^ Whereas another study didn't discern differences between these two states in breast tissues, which also applied limma package of R, but with the significance of false discovery rate adjusted *p* ≤ 0.01 and fold change ≥ 2.0,^54^ and this may partly explain the inconsistency. In addition, though the original study^19^ found no gene expression differences by unsupervised hierarchical clustering or principal component analysis of all genes, we were not sure if the aforesaid reason was included because the concrete cutoff of significance did not elucidate. Therefore, the gene expression perturbations in para‐cancerous tissues are hard to be determined, which might depend on how far away from the tumor to get the tissue, analytical methods used, and a certain number of samples. For DEGs between BC and para‐cancerous or normal tissues, we will not discuss them here, since they have been extensively studied.

In fact, *JUN*, *FOS*, *FOSB*, and *ATF3* are components of TF activator protein‐1 (AP‐1), a type of dimeric TF that has been widely studied and is formed by JUN, FOS, ATF, and MAF protein families, which regulates a variety of functions, such as cell proliferation, differentiation, transformation, apoptosis, survival,^55^, ^56^ migration, and oncogenesis in the response to extracellular and external stimuli such as cytokines, growth factors, stress signals, bacterial and viral infections, and oncoproteins.^57^, ^58^ Moreover, previous studies concluded that AP‐1 was mainly associated with autoimmune disorders, carcinomas, and hematological malignancies.^58^ Initially, proteins of the AP‐1 family, JUN, FOS, and ATF were considered to be oncogenic, whereafter JUNB and FOS were reported to suppress tumorigenesis.^59^ JUN was then illustrated to suppress tumorigenesis.^60^ On the other hand, some studies indicated that AP‐1 might play important roles in tumor cell invasion^61^, ^62^ and metastasis.^63^ These studies as well confirmed different effects of *FOS* on the tumor.

In BC, previous studies found that *FOS* and *JUN* were downregulated in BC compared with adjacent and normal tissues,^64^, ^65^ but *FOSB* had no difference.^65^ In other studies, *FOS* and *FOSB* were found to increase cancer cell invasion,^61^ and *JUN* was involved in cancer cell invasion and metastasis.^66^ Recently, researchers have further elucidated the mechanisms of their functions in tumorigenesis, tumor cell invasion, and metastasis. For instance, Xie et al.^67^ reported that *JUN* was upregulated in TNBC than non‐TNBC tumors, and the JUN N‐terminal kinase (JNK)/JUN signaling pathway might regulate cancer stem‐like cell phenotype and tumorigenesis through Notch1 signaling pathway in TNBC, while Anders et al.^68^ demonstrated that hyper‐phosphorylation of JUN by JNK inhibited tumor cell migration and invasion. Diann. et al. found that *FOS* promoted the expression of *CRADD* and *UQCRC2*, two apoptosis‐effector genes, and was associated with better survival outcomes.^69^ A recent study found that *FOSB* was significantly decreased in different subtypes of BC samples. Its higher expression was related to better RFS in TNBC and Her2‐ BC based on Oncomine and TCGA datasets, and increased TNBC cell proliferation in the *FOSB* knockout model conversely indicated its ability of tumor inhibition.^70^ Furthermore, studies on drug‐treated TNBC cells showed that FOSB might be a pro‐apoptotic protein and led to BC cell death,^71^ whereas the upregulated *FOS* in response to eribulin treatment in TNBC cells might be responsible for the low drug sensitivity.^72^ *ATF3* was highly expressed when the organism encountered cancer cells as an adaptive‐response gene, which could induce apoptosis as a tumor suppressor or enhance BC cell tumorigenicity as an oncogene, contributing to epithelial‐to‐mesenchymal transition and metastasis.^73^, ^74^ A current research showed that *ATF3* was downregulated in BC compared with adjacent tissues, and overexpression of which enhanced BC cell apoptosis and suppress cell growth, migration, and invasion.^75^

Early growth response 1 (*EGR1*) was considered as a tumor suppressor regulating the expressions of several cancer suppressor genes, including *TGFβ1*, *IGF*‐*II*, *PTEN*, *fibronectin*, *p53*, and *p73*, while in prostate cancer as a tumor enhancer.^76^ The low expression of *EGR1* led to poor prognosis and therapeutic resistance.^77^ Human *IER2* was upregulated in plenty of primary tumors compared with paired adjacent normal tissues including BC,^78^ whereas it was downregulated in hepatocellular carcinoma compared with matched normal tissues, but enhanced hepatocellular carcinoma cell motility and metastasis.^79^ *ZFP36* is downregulated in the tumor compared with normal samples, and co‐expressed with *JUN*, *JUNB*, *FOS*, and *FOSB*, downregulation of which was associated with poor outcomes and BC progression. Thereby, *ZFP36* was regarded as a tumor suppressor, and several non‐coding RNAs could restrain tumor growth through it.^80^, ^81^ Nuclear receptor subfamily four group A member 2 (*NR4A2*) were involved in cell proliferation, apoptosis, inflammation, and cancer,^82^ and highly expressed in CD133^+^ colorectal carcinoma cells, which could be a tumor suppressor or promoter depending on the tumor provenance,^83^ and no research linked it to BC yet. However, little emphasis was put on the mechanisms of these low‐expressed genes in para‐cancerous compared with normal tissues. Therefore, our findings provided more evidence to lay a foundation for that.

PPARs, a kind of transcriptional factors belonging to nuclear receptor superfamily, are made up of PPAR‐a, PPAR‐b/d, and PPAR‐g, which are associated with adipocyte differentiation, lipid metabolism, hyperlipidemia, insulin sensitization, cancer, inflammation, and atherosclerosis.^84^ According to KEGG pathway enrichment and module function analyses, the PPAR signaling pathway was significantly enriched in our research model. A recent study^12^ combining four GEO and TCGA databases of BC also identified PPAR signaling pathway as a main pathway involved in BC, indicating that the PPAR signaling pathway‐regulated BC progression. As Figure 4F showed that seven module genes were gathered in PPAR pathway, namely *PCK1*, *PPARG*, *PLIN1*, *ADIPOQ*, *FABP4*, *LPL*, and *ACSL1*, of which *PPARG* showed the highest degrees in the PPI network and the upstream location in this pathway (Figure S11). PPARG constitutes heterodimer with retinoid X receptor and thus conducts transcription of multiple genes, which mainly regulates adipocyte differentiation and is related to cancer, inflammation, and atherosclerosis.^84^ In BC, researches indicated that PPARG inhibited tumor progression^85^, ^86^, ^87^ as a promising antineoplastic agent in the clinic.^88^

Complement factor D (*CFD*), also known as *ADIPSIN*, targeted by *PPARG*,^89^ is secreted from adipocytes. It was reported to be expressed in adipose tissues surrounding a tumor, which could promote BC cell growth and cancer stem cell‐like properties.^90^ *CD27* is an immune checkpoint gene,^91^ whose signal contributes to antitumor immunity in humans. Ongoing clinical trial targeting *CD27* has been established.^92^ *PSMB9*, a kind of immunoproteasome gene, consistent with our findings, was reported to be highly expressed in BC and associated with long survival.^93^ Selectin L (*SELL*) was also highly expressed in BC and associated with better outcomes of BC patients according to public data.^94^

In summary, our work implemented a comprehensive bioinformatic analysis on BC, showing different transcriptional variations in para‐cancerous tissues and BC. Our findings deepened our understanding of the mechanisms of tumorigenesis, invasion, relapse, progression, and prognosis of BC, and provided promisingly diagnostic and therapeutic targets for BC patients.

## CONFLICT OF INTEREST

The authors declare no conflict of interest.

## AUTHOR CONTRIBUTION

Xingxing Dong, Yan Gong, and Gaosong Wu conceived the study; Xingxing Dong, Gaoran Xu, and Zelin Tian delt with the data; Xingxing Dong and Qian Yang performed the visualization; Xingxing Dong wrote the draft; Yalong Yang, Yan Gong, and Gaosong Wu revised the paper. All authors have read and agreed to the published version of the manuscript.

## ETHICS STATEMENT

All studies involving human samples have received the patient's informed consent and the approval of the Wuhan University Ethics Committee.

## Supporting information

Supplementary Material

## Data Availability

The data presented in this study are openly available in GEO database (https://www.ncbi.nlm.nih.gov/geo/), reference number.^18^, ^62^, ^63^

## References

[1] Sung H , et al. Global cancer statistics 2020: GLOBOCAN estimates of incidence and mortality worldwide for 36 cancers in 185 countries. CA Cancer J Clin. 2021;71(3):209‐249. 33538338 10.3322/caac.21660

[2] Aleskandarany MA , Vandenberghe ME , Marchiò C , et al. Tumour heterogeneity of breast cancer: from morphology to personalised medicine. Pathobiology. 2018;85(1–2):23‐34. 29428954 10.1159/000477851

[3] Dai X , Li T , Bai Z , et al. Breast cancer intrinsic subtype classification, clinical use and future trends. Am J Cancer Res. 2015;5(10):2929‐2943. 26693050 PMC4656721

[4] Harbeck N , Penault‐Llorca F , Cortes J , et al. Breast cancer. Nat Rev Dis Primers. 2019;5(1):66. 31548545 10.1038/s41572-019-0111-2

[5] Waks AG , Winer EP . Breast cancer treatment: a review. JAMA. 2019;321(3):288‐300. 30667505 10.1001/jama.2018.19323

[6] DeSantis CE , Ma J , Gaudet MM , et al. Breast cancer statistics, 2019. CA Cancer J Clin. 2019;69(6):438‐451. 31577379 10.3322/caac.21583

[7] Schmid P , Adams S , Rugo HS , et al. Atezolizumab and Nab‐paclitaxel in advanced triple‐negative breast cancer. N Engl J Med. 2018;379(22):2108‐2121. 30345906 10.1056/NEJMoa1809615

[8] Zhang H . Overview of sequence data formats. Methods Mol Biol. 2016;1418:3‐17. 27008007 10.1007/978-1-4939-3578-9_1

[9] Gentleman R , Carey VJ , Huber W , et al. Bioinformatics and Computational Biology Solutions Using R and Bioconductor. Springer; 2005.

[10] Zeng Q , et al. Identification of therapeutic targets and prognostic biomarkers among CXC chemokines in the renal cell carcinoma microenvironment. Front Oncol. 2019;9:1555. 32117786 10.3389/fonc.2019.01555 PMC7012904

[11] Cui X , Zhang X , Liu M , et al. A pan‐cancer analysis of the oncogenic role of staphylococcal nuclease domain‐containing protein 1 (SND1) in human tumors. Genomics. 2020;112(6):3958‐3967. 32645525 10.1016/j.ygeno.2020.06.044

[12] Xu Y‐H , Deng J‐L , Wang L‐P , et al. Identification of candidate genes associated with breast cancer prognosis. DNA Cell Biol. 2020;39(7):1205‐1227. 32456464 10.1089/dna.2020.5482

[13] Meng L‐B , Shan M‐J , Qiu Y , et al. TPM2 as a potential predictive biomarker for atherosclerosis. Aging. 2019;11:6960‐6982. 31487691 10.18632/aging.102231 PMC6756910

[14] Ge W‐H , Lin Y , Li S , et al. Identification of biomarkers for early diagnosis of acute myocardial infarction. J Cell Biochem. 2018;119(1):650‐658. 28636181 10.1002/jcb.26226

[15] Li L , Lei Q , Zhang S , et al. Screening and identification of key biomarkers in hepatocellular carcinoma: evidence from bioinformatic analysis. Oncol Rep. 2017;38(5):2607‐2618. 28901457 10.3892/or.2017.5946 PMC5780015

[16] Guo Y , Bao Y , Ma M , et al. Identification of key candidate genes and pathways in colorectal cancer by integrated bioinformatical analysis. Int J Mol Sci. 2017;18(4):722. 10.3390/ijms18040722 PMC5412308 28350360

[17] Wang J , Wang Y , Kong F , et al. Identification of a six‐gene prognostic signature for oral squamous cell carcinoma. J Cell Physiol. 2020;235(3):3056‐3068. 31538341 10.1002/jcp.29210

[18] Clough E , Barrett T . The gene expression omnibus database. Methods Mol Biol. 2016;1418:93‐110. 27008011 10.1007/978-1-4939-3578-9_5 PMC4944384

[19] Tripathi A , King C , de la Morenas A , et al. Gene expression abnormalities in histologically normal breast epithelium of breast cancer patients. Int J Cancer. 2008;122(7):1557‐1566. 18058819 10.1002/ijc.23267

[20] Pau Ni IB , Zakaria Z , Muhammad R , et al. Gene expression patterns distinguish breast carcinomas from normal breast tissues: the Malaysian context. Pathol Res Pract. 2010;206(4):223‐228. 20097481 10.1016/j.prp.2009.11.006

[21] Clarke C , Madden SF , Doolan P , et al. Correlating transcriptional networks to breast cancer survival: a large‐scale coexpression analysis. Carcinogenesis. 2013;34(10):2300‐2308. 23740839 10.1093/carcin/bgt208

[22] Heber S , Sick B . Quality assessment of Affymetrix GeneChip data. OMICS J Integr Biol. 2006;10(3):358‐368. 10.1089/omi.2006.10.358 17069513

[23] Irizarry RA , et al. Summaries of Affymetrix GeneChip probe level data. Nucleic Acids Res. 2003;31(4):e15. 12582260 10.1093/nar/gng015 PMC150247

[24] Troyanskaya O , Cantor M , Sherlock G , et al. Missing value estimation methods for DNA microarrays. Bioinformatics. 2001;17(6):520‐525. 11395428 10.1093/bioinformatics/17.6.520

[25] Smyth GK . Limma: Linear Models for Microarry Data. Springer. 2005;397‐420.

[26] Gene Ontology Consortium . The gene ontology resource 20 years and still GOing strong. Nucleic Acids Res. 2018;47:D330‐D338. 10.1093/nar/gky1055 PMC6323945 30395331

[27] Consortium , Ashburner M , Ball CA , Blake JA , et al. Gene Ontology: tool for the unification of biology. Nat Genet. 2000;25:25‐29. 10802651 10.1038/75556 PMC3037419

[28] Kanehisa M , Sato Y , Furumichi M , et al. New approach for understanding genome variations in KEGG. Nucleic Acids Res. 2019;47(D1):D590‐D595. 30321428 10.1093/nar/gky962 PMC6324070

[29] Szklarczyk D , Gable AL , Lyon D , et al. STRING v11: protein‐protein association networks with increased coverage, supporting functional discovery in genome‐wide experimental datasets. Nucleic Acids Res. 2019;47(D1):D607‐D613. 30476243 10.1093/nar/gky1131 PMC6323986

[30] Zhang B , Horvath S . A general framework for weighted gene co‐expression network analysis. Stat Appl Genet Mol Biol. 2005;4(1). 10.2202/1544-6115.1128 16646834

[31] Langfelder P , Horvath S . WGCNA: an R package for weighted correlation network analysis. BMC Bioinformatics. 2008;9:559. 19114008 10.1186/1471-2105-9-559 PMC2631488

[32] Smoot ME , Ono K , Ruscheinski J , et al. Cytoscape 2.8: new features for data integration and network visualization. Bioinformatics. 2011;27(3):431‐432. 21149340 10.1093/bioinformatics/btq675 PMC3031041

[33] Bader GD , Hogue CW . An automated method for finding molecular complexes in large protein interaction networks. BMC Bioinformatics. 2003;4(2):1471‐2105. 10.1186/1471-2105-4-2 PMC149346 12525261

[34] Zhou Y , Zhou B , Pache L , et al. Metascape provides a biologist‐oriented resource for the analysis of systems‐level datasets. Nat Commun. 2019;10(1):1523. 30944313 10.1038/s41467-019-09234-6 PMC6447622

[35] Han H , Cho J‐W , Lee S , et al. TRRUST v2: an expanded reference database of human and mouse transcriptional regulatory interactions. Nucleic Acids Res. 2018;46(D1):D380‐D386. 29087512 10.1093/nar/gkx1013 PMC5753191

[36] Chandrashekar DS , Bashel B , Balasubramanya SAH , et al. UALCAN: a portal for facilitating tumor subgroup gene expression and survival analyses. Neoplasia. 2017;19(8):649‐658. 28732212 10.1016/j.neo.2017.05.002 PMC5516091

[37] Tomczak K , Czerwinska P , Wiznerowicz M . The Cancer Genome Atlas (TCGA): an immeasurable source of knowledge. Contemp Oncol (Pozn). 2015;19(1A):A68‐A77. 25691825 10.5114/wo.2014.47136 PMC4322527

[38] Jézéquel P , Campone M , Gouraud W , et al. bc‐GenExMiner: an easy‐to‐use online platform for gene prognostic analyses in breast cancer. Breast Cancer Res Treat. 2012;131(3):765‐775. 21452023 10.1007/s10549-011-1457-7

[39] Jezequel P , Frenel J‐S , Campion L , et al. bc‐GenExMiner 3.0: new mining module computes breast cancer gene expression correlation analyses. Database (Oxford). 2013;2013:bas060. 23325629 10.1093/database/bas060 PMC3548333

[40] Li T , Fu J , Zeng Z , et al. TIMER2.0 for analysis of tumor‐infiltrating immune cells. Nucleic Acids Res. 2020;48(W1):W509‐W514. 32442275 10.1093/nar/gkaa407 PMC7319575

[41] Cerami E , et al. The cBio cancer genomics portal: an open platform for exploring multidimensional cancer genomics data. Cancer Discov. 2012;2(5):401‐404. 22588877 10.1158/2159-8290.CD-12-0095 PMC3956037

[42] Subramanian A , Tamayo P , Mootha VK , et al. Gene set enrichment analysis: a knowledge‐based approach for interpreting genome‐wideexpression profiles. Proc Natl Acad Sci USA. 2005;102(43):15545‐15550. 16199517 10.1073/pnas.0506580102 PMC1239896

[43] Pilewskie M , Morrow M . Margins in breast cancer: how much is enough? Cancer. 2018;124(7):1335‐1341. 29338088 10.1002/cncr.31221 PMC5894883

[44] Lambert SA , Jolma A , Campitelli LF , et al. The human transcription factors. Cell. 2018;172(4):650‐665. 29425488 10.1016/j.cell.2018.01.029 PMC12908702

[45] Nagarajan D , McArdle SEB . Immune landscape of breast cancers. Biomedicines. 2018;6:1. 10.3390/biomedicines6010020 PMC5874677 29439457

[46] Schreiber RD , Old LJ , Smyth MJ . Cancer immunoediting: integrating immunity's roles in cancer suppression and promotion. Science. 2011;331(6024):1565‐1570. 21436444 10.1126/science.1203486

[47] Savas P , Salgado R , Denkert C , et al. Clinical relevance of host immunity in breast cancer: from TILs to the clinic. Nat Rev Clin Oncol. 2016;13(4):228‐241. 26667975 10.1038/nrclinonc.2015.215

[48] Gao G , Wang Z , Qu X , et al. Prognostic value of tumor‐infiltrating lymphocytes in patients with triple‐negative breast cancer: a systematic review and meta‐analysis. BMC Cancer. 2020;20(1):179. 32131780 10.1186/s12885-020-6668-z PMC7057662

[49] Ishii G , Ochiai A , Neri S . Phenotypic and functional heterogeneity of cancer‐associated fibroblast within the tumor microenvironment. Adv Drug Deliv Rev. 2016;99(Pt B):186‐196. 26278673 10.1016/j.addr.2015.07.007

[50] Xia Y , Rao L , Yao H , et al. Engineering macrophages for cancer immunotherapy and drug delivery. Adv Mater. 2020;32(40):e2002054. 32856350 10.1002/adma.202002054

[51] Esteller M . CpG island hypermethylation and tumor suppressor genes: a booming present, a brighter future. Oncogene. 2002;21(35):5427‐5440. 12154405 10.1038/sj.onc.1205600

[52] Kuscu C , Evensen N , Kim D , et al. Transcriptional and epigenetic regulation of KIAA1199 gene expression in human breast cancer. PLoS One. 2012;7(9):e44661. 22970280 10.1371/journal.pone.0044661 PMC3435267

[53] Abdalla M , Tran‐Thanh D , Moreno J , et al. Mapping genomic and transcriptomic alterations spatially in epithelial cells adjacent to human breast carcinoma. Nat Commun. 2017;8(1):1245. 29093438 10.1038/s41467-017-01357-y PMC5665998

[54] Finak G , Sadekova S , Pepin F , et al. Gene expression signatures of morphologically normal breast tissue identify basal‐like tumors. Breast Cancer Res. 2006;8(5):R58. 17054791 10.1186/bcr1608 PMC1779486

[55] Hess J , Angel P , Schorpp‐Kistner M . AP‐1 subunits: quarrel and harmony among siblings. J Cell Sci. 2004;117(Pt 25):5965‐5973. 15564374 10.1242/jcs.01589

[56] Shaulian E , Karin M . AP‐1 as a regulator of cell life and death. Nat Cell Biol. 2002;4:131‐136. 10.1038/ncb0502-e131 11988758

[57] Lopez‐Bergami P , Lau E , Ronai Z . Emerging roles of ATF2 and the dynamic AP1 network in cancer. Nat Rev Cancer. 2010;10(1):65‐76. 20029425 10.1038/nrc2681 PMC2874064

[58] Trop‐Steinberg S , Azar Y . AP‐1 expression and its clinical relevance in immune disorders and cancer. Am J Med Sci. 2017;353(5):474‐483. 28502334 10.1016/j.amjms.2017.01.019

[59] Eferl R , Wagner EF . AP‐1: a double‐edged sword in tumorigenesis. Nat Rev Cancer. 2003;3(11):859‐868. 14668816 10.1038/nrc1209

[60] Shaulian E . AP‐1–The Jun proteins: oncogenes or tumor suppressors in disguise? Cell Signal. 2010;22(6):894‐899. 20060892 10.1016/j.cellsig.2009.12.008

[61] Ozanne BW , Spence HJ , McGarry LC , et al. Transcription factors control invasion: AP‐1 the first among equals. Oncogene. 2007;26(1):1‐10. 16799638 10.1038/sj.onc.1209759

[62] Vleugel MM , Greijer AE , Bos R , et al. c‐Jun activation is associated with proliferation and angiogenesis in invasive breast cancer. Hum Pathol. 2006;37(6):668‐674. 16733206 10.1016/j.humpath.2006.01.022

[63] Zhang Y , et al. Critical role of c‐Jun overexpression in liver metastasis of human breast cancer xenograft model. BMC Cancer. 2007;7:145. 17672916 10.1186/1471-2407-7-145 PMC1959235

[64] Smith LM , Birrer MJ , Stampfer MR , Brown PH . Breast cancer cells have lower activity protein 1 transcription factor activity than normal mammary epithelial cells. Can Res. 1997;57:3046‐3054. 9230221

[65] Kharman‐Biz A , Gao H , Ghiasvand R , et al. Expression of activator protein‐1 (AP‐1) family members in breast cancer. BMC Cancer. 2013;13:441. 24073962 10.1186/1471-2407-13-441 PMC3849565

[66] Zhang Y , Pu X , Shi M , et al. c‐Jun, a crucial molecule in metastasis of breast cancer and potential target for biotherapy. Oncol Rep. 2007;18(5):1207‐1212. 17914574

[67] Xie X , Kaoud TS , Edupuganti R , et al. c‐Jun N‐terminal kinase promotes stem cell phenotype in triple‐negative breast cancer through upregulation of Notch1 via activation of c‐Jun. Oncogene. 2017;36(18):2599‐2608. 27941886 10.1038/onc.2016.417 PMC6116358

[68] Sundqvist A , et al. JNK‐dependent cJun phosphorylation mitigates TGFbeta‐ and EGF‐induced pre‐malignant breast cancer cell invasion by suppressing AP‐1‐mediated transcriptional responses. Cells. 2019;8(12):1481. 10.3390/cells8121481 PMC6952832 31766464

[69] Fisler D , Sikaria D , Yavorski J , et al. Elucidating feed‐forward apoptosis signatures in breast cancer datasets: higher FOS expression associated with a better outcome. Oncol Lett. 2018;16(2):2757‐2763. 30013671 10.3892/ol.2018.8957 PMC6036554

[70] Zhang R , Li X , Liu Z , et al. EZH2 inhibitors‐mediated epigenetic reactivation of FOSB inhibits triple‐negative breast cancer progress. Cancer Cell Int. 2020;20:175. 32477007 10.1186/s12935-020-01260-5 PMC7236314

[71] Park JA , Na H‐H , Jin H‐O , Kim K‐C . Increased expression of FosB through reactive oxygen species accumulation functions as pro‐apoptotic protein in piperlongumine treated MCF7 breast cancer cells. Mol Cells. 2019;42(12):884‐892. 31735020 10.14348/molcells.2019.0088 PMC6939652

[72] Tanaka S , Ishii T , Sato F , et al. Eribulin mesylate‐induced c‐Fos upregulation enhances cell survival in breast cancer cell lines. Biochem Biophys Res Comm. 2020;526(1):154‐157. 32201082 10.1016/j.bbrc.2020.03.042

[73] Yin X , Wolford CC , Chang Y‐S et al. ATF3, an adaptive‐response gene, enhances TGF beta signaling and cancer‐initiating cell features in breast cancer cells. J Cell Sci. 2010;123(20):3558‐3565. 20930144 10.1242/jcs.064915 PMC2951469

[74] Wolford CC , McConoughey SJ , Jalgaonkar SP , et al. Transcription factor ATF3 links host adaptive response to breast cancer metastasis. J Clin Invest. 2013;123(7):2893‐2906. 23921126 10.1172/JCI64410 PMC3696548

[75] Li LQ , Sun RM , Jiang GQ . ATF3 demethylation promotes the transcription of ARL4C, which acts as a tumor suppressor in human breast cancer. Onco Targets Ther. 2020;13:3467‐3476. 32425548 10.2147/OTT.S243632 PMC7195577

[76] Baron V , Adamson ED , Calogero A , Ragona G , Mercola D . The transcription factor Egr1 is a direct regulator of multiple tumor suppressors including TGFβ1, PTEN, p53 and fibronectin: Egr1 is a potential target of gene therapy for prostate cancer. Cancer Gene Ther. 2006;13(2):115‐124. 16138117 10.1038/sj.cgt.7700896 PMC2455793

[77] Shajahan‐Haq AN , Boca SM , Jin LU , et al. EGR1 regulates cellular metabolism and survival in endocrine resistant breast cancer. Oncotarget. 2017;8(57):96865‐96884. 29228577 10.18632/oncotarget.18292 PMC5722529

[78] Neeb A , Wallbaum S , Novac N , et al. The immediate early gene Ier2 promotes tumor cell motility and metastasis, and predicts poor survival of colorectal cancer patients. Oncogene. 2012;31(33):3796‐3806. 22120713 10.1038/onc.2011.535

[79] Xu X , Zhou W , Chen Y , et al. Immediate early response protein 2 promotes the migration and invasion of hepatocellular carcinoma cells via regulating the activity of Rho GTPases. Neoplasma. 2020;67(3):614‐622. 32009420 10.4149/neo_2020_190818N781

[80] Canzoneri R , Naipauer J , Stedile M , et al. Identification of an AP1‐ZFP36 regulatory network associated with breast cancer prognosis. J Mammary Gland Biol Neoplasia. 2020;25(2):163‐172. 32248342 10.1007/s10911-020-09448-1

[81] Mao Y , Lv M , Cao W , et al. Circular RNA 000554 represses epithelial‐mesenchymal transition in breast cancer by regulating microRNA‐182/ZFP36 axis. FASEB J. 2020;34(9):11405‐11420. 32729957 10.1096/fj.201903047R

[82] Yamamoto S , Komiya T . PS01.32: high throughput screening of small molecule Inhibitors for Nuclear Receptor Subfamily 4 Group A Member 2 (NR4A2) in human cancers. J Thorac Oncol. 2016;11(11):S288‐S289.

[83] Han YF , Cao GW . Role of nuclear receptor NR4A2 in gastrointestinal inflammation and cancers. World J Gastroenterol. 2012;18(47):6865‐6873. 23322982 10.3748/wjg.v18.i47.6865 PMC3531668

[84] Kota BP , Huang THW , Roufogalis BD . An overview on biological mechanisms of PPARs. Pharmacol Res. 2005;51(2):85‐94. 15629253 10.1016/j.phrs.2004.07.012

[85] Yang PB , et al. Blocking PPAR gamma interaction facilitates Nur77 interdiction of fatty acid uptake and suppresses breast cancer progression. Proc Natl Acad Sci USA. 2020;117(44):27412‐27422. 33087562 10.1073/pnas.2002997117 PMC7959534

[86] Xu YY , et al. PPAR gamma inhibits breast cancer progression by upregulating PTPRF expression. Eur Rev Med Pharmacol Sci. 2019;23(22):9965‐9977. 31799666 10.26355/eurrev_201911_19563

[87] Lightbody ED , et al. PPAR gamma loss increases the metastatic potential of HER2^+^ breast cancer. Can Res. 2018;78(13).

[88] Augimeri G , et al. The role of PPAR gamma ligands in breast cancer: from basic research to clinical studies. Cancers. 2020;12(9):2623. 10.3390/cancers12092623 PMC7564201 32937951

[89] Brown JD , Plutzky J . Peroxisome proliferator‐activated receptors as transcriptional nodal points and therapeutic targets. Circulation. 2007;115(4):518‐533. 17261671 10.1161/CIRCULATIONAHA.104.475673

[90] Goto H , Shimono Y , Funakoshi Y , et al. Adipose‐derived stem cells enhance human breast cancer growth and cancer stem cell‐like properties through adipsin. Oncogene. 2019;38(6):767‐779. 30177835 10.1038/s41388-018-0477-8

[91] Fang J , Chen F , Liu D , et al. Prognostic value of immune checkpoint molecules in breast cancer. Biosci Rep. 2020;40(7):1‐13. 10.1042/BSR20201054 PMC7340863 32602545

[92] Buchan SL , Rogel A , Al‐Shamkhani A . The immunobiology of CD27 and OX40 and their potential as targets for cancer immunotherapy. Blood. 2018;131(1):39‐48. 29118006 10.1182/blood-2017-07-741025

[93] Rouette A , et al. Expression of immunoproteasome genes is regulated by cell‐intrinsic and ‐extrinsic factors in human cancers. Sci Rep. 2016;6:34019. 27659694 10.1038/srep34019 PMC5034284

[94] Kumari S , Arora M , Singh J , et al. L‐Selectin expression is associated with inflammatory microenvironment and favourable prognosis in breast cancer. 3 Biotech. 2021;11(2):38. 10.1007/s13205-020-02549-y PMC7794266 33479593

