# Supplementary material for: The initial expression alterations occurring to transcription factors during the formation of breast cancer: Evidence from bioinformatics
Source: Cancer Med. 2022 Jan 17;11(5):1371–95. doi: 10.1002/cam4.4545 (PMC8894706; doi:10.1002/cam4.4545)
Supplement: Supplementary file 1 — Supplementary Material [file CAM4-11-1371-s001.pdf]

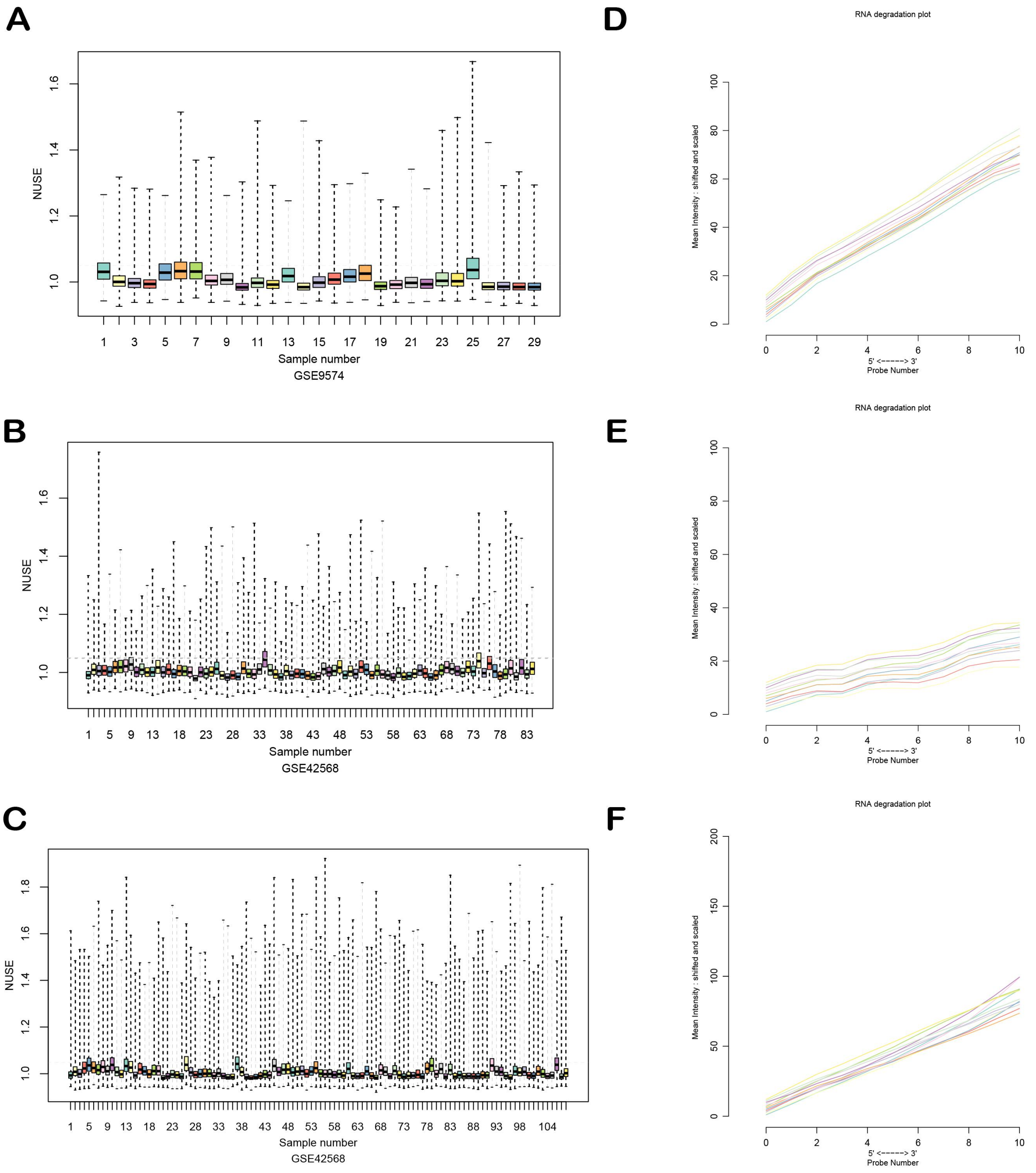

Figure S1. Raw data quality control at probe level. (**A-C**) Normalized unscaled standard error (NUSE) boxplots for GSE9574, GSE15852 and GSE42568, each box of which represents a gene chip or sample. The gray dotted line corresponds to the alarming value of 1.05. (**D-F**) RNA degradation plots of GSE9574, GSE15852 and GSE42568, each line in which represents the degradation of a sample.

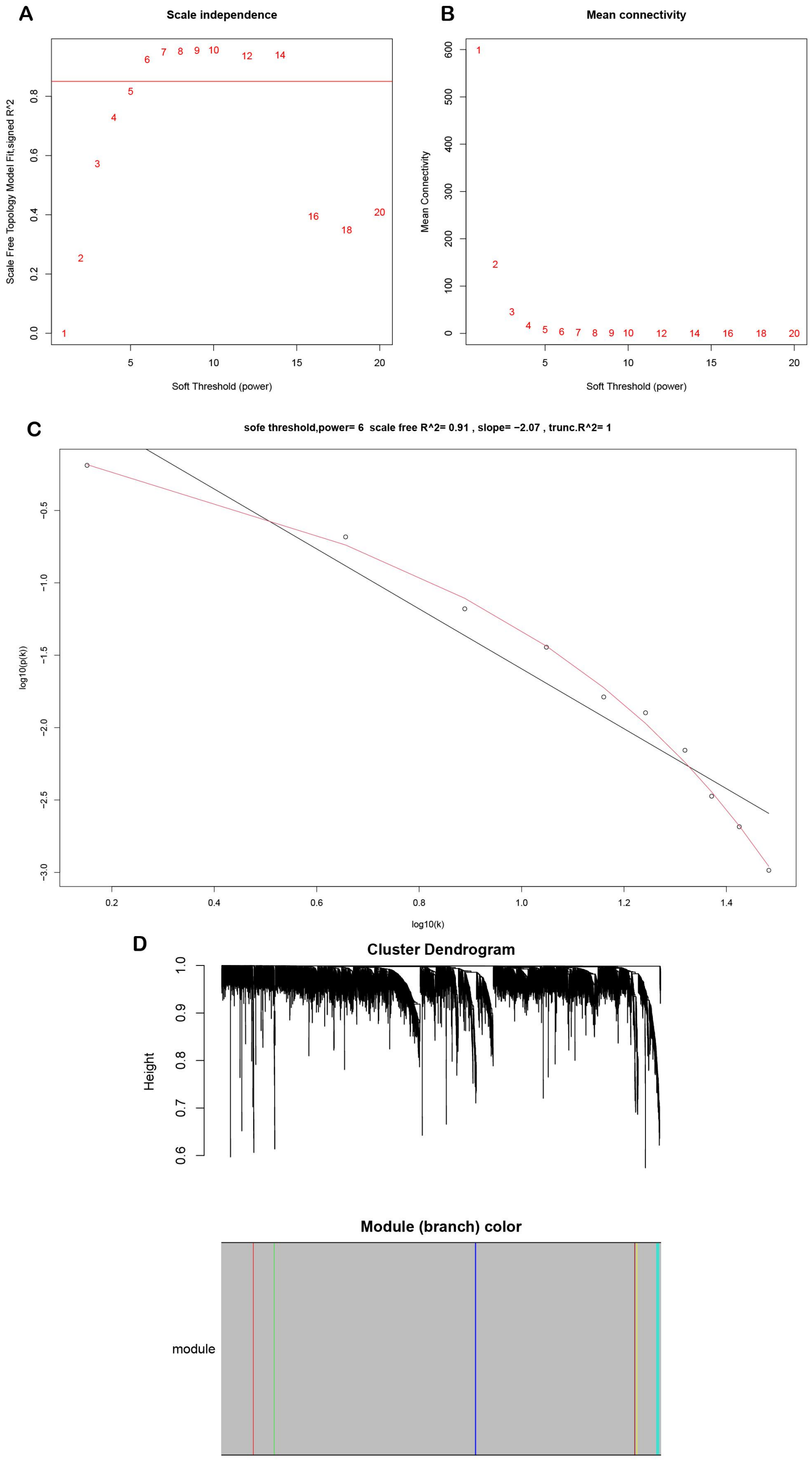

Figure S2. WGCNA performed on 94 BC samples in GSE42568. (**A**) The corresponding signed R^2 (scale-free topology fit) of different soft thresholds (powers). The red line represents signed $R^2 = 0.85$ and a network with signed $R^2 > 0.8$ could be considered to meet scale-free topology. (**B**) The mean connectivity under different soft thresholds. (**C**) Scale-free topology plot of the network that was built with the soft threshold = 6. The scale-free R2 = 0.91 indicated that the network satisfied scale-free topology. (**D**) Clustering dendrogram and six detected modules in the network. Different colors represent different modules.

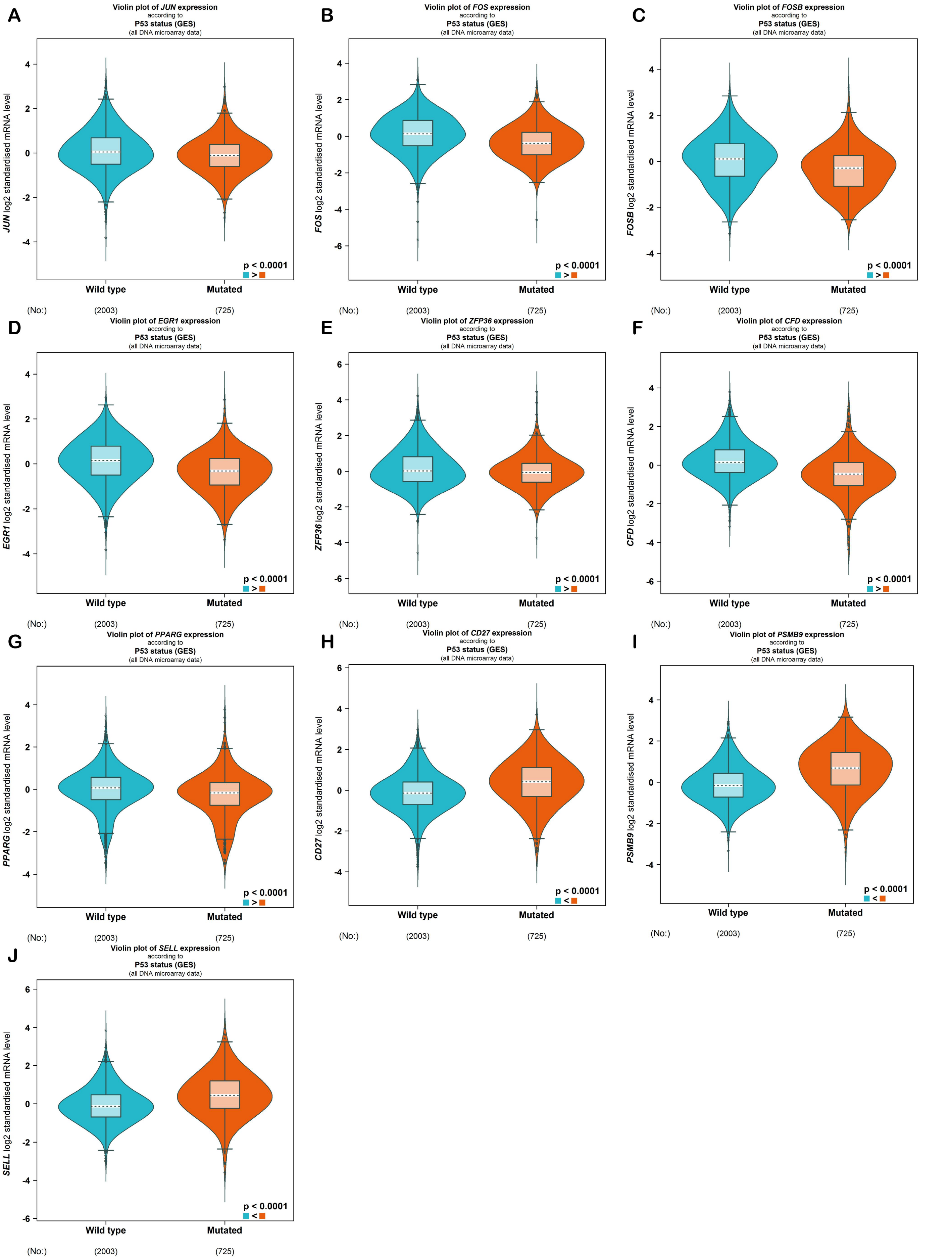

Figure S3. Expression levels of candidate genes based on TP53 mutation status in BC. Violin plots with p value < 0.05 are presented.

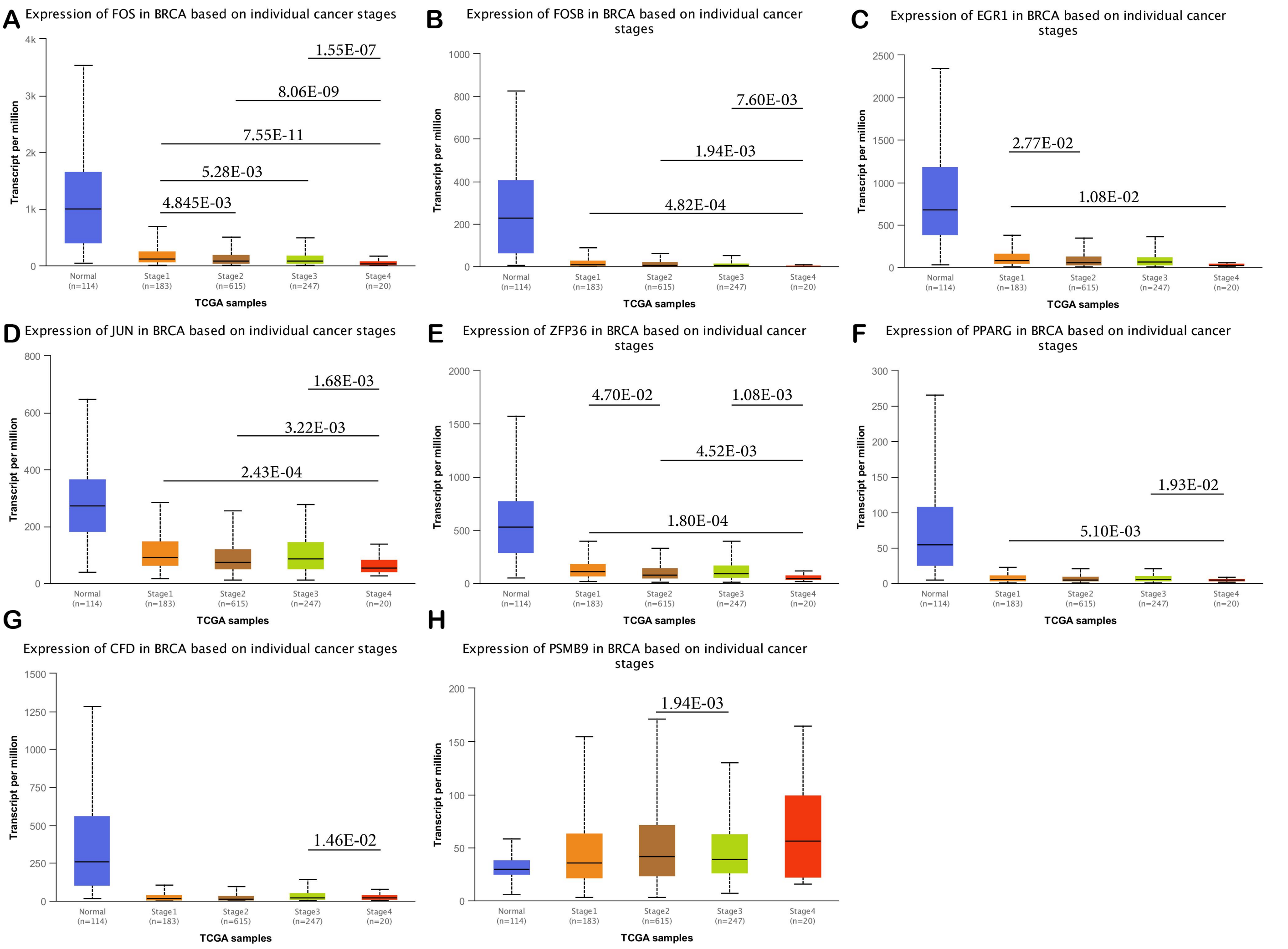

Figure S4. Expression levels of candidate genes based on individual cancer stage of BC patients. Boxplots with p value < 0.05 between at least two stages are presented, as well as the corresponding p value.

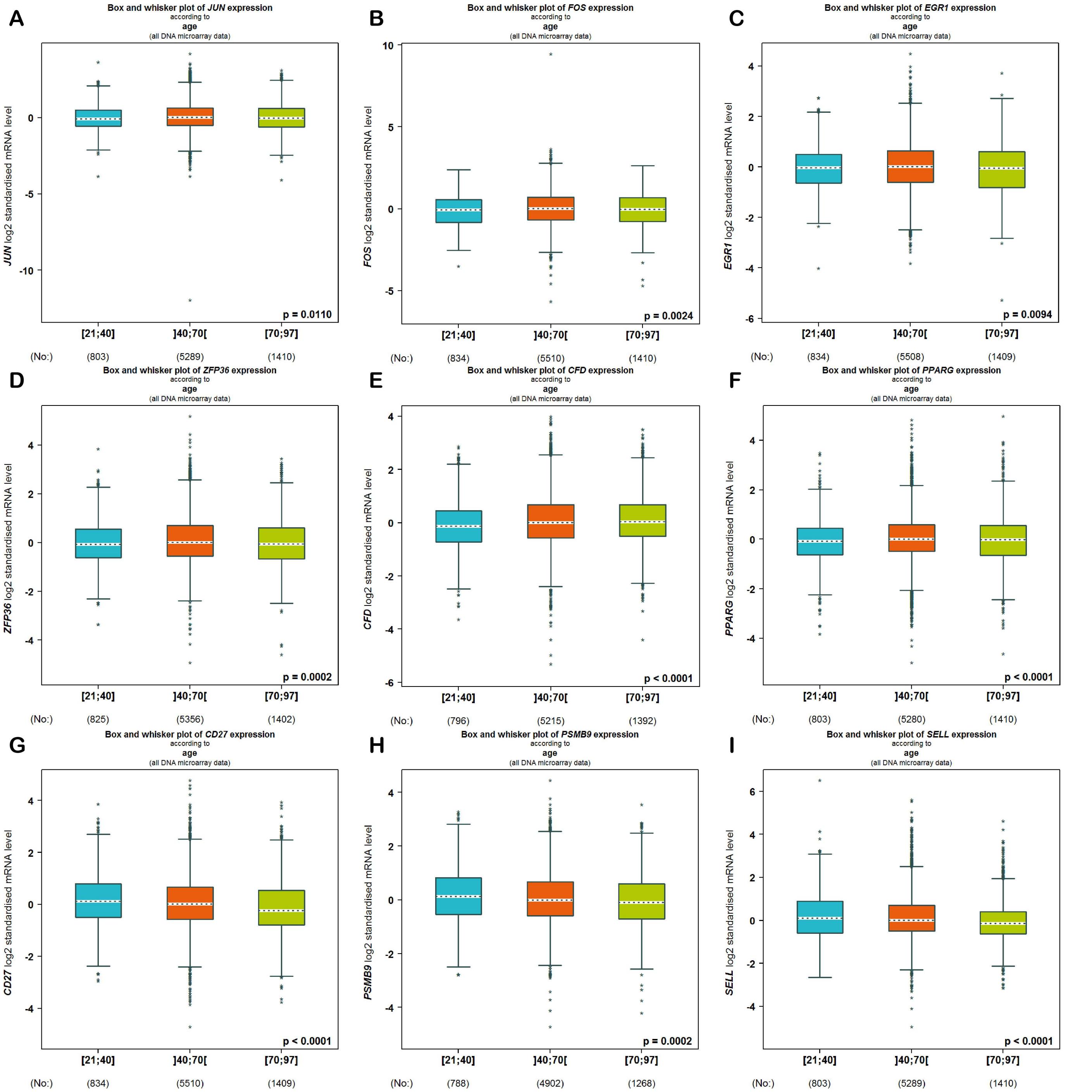

Figure S5. Expression levels of candidate genes based on BC patients' age. Boxplots with p value < 0.05 are presented, as well as the corresponding p value.

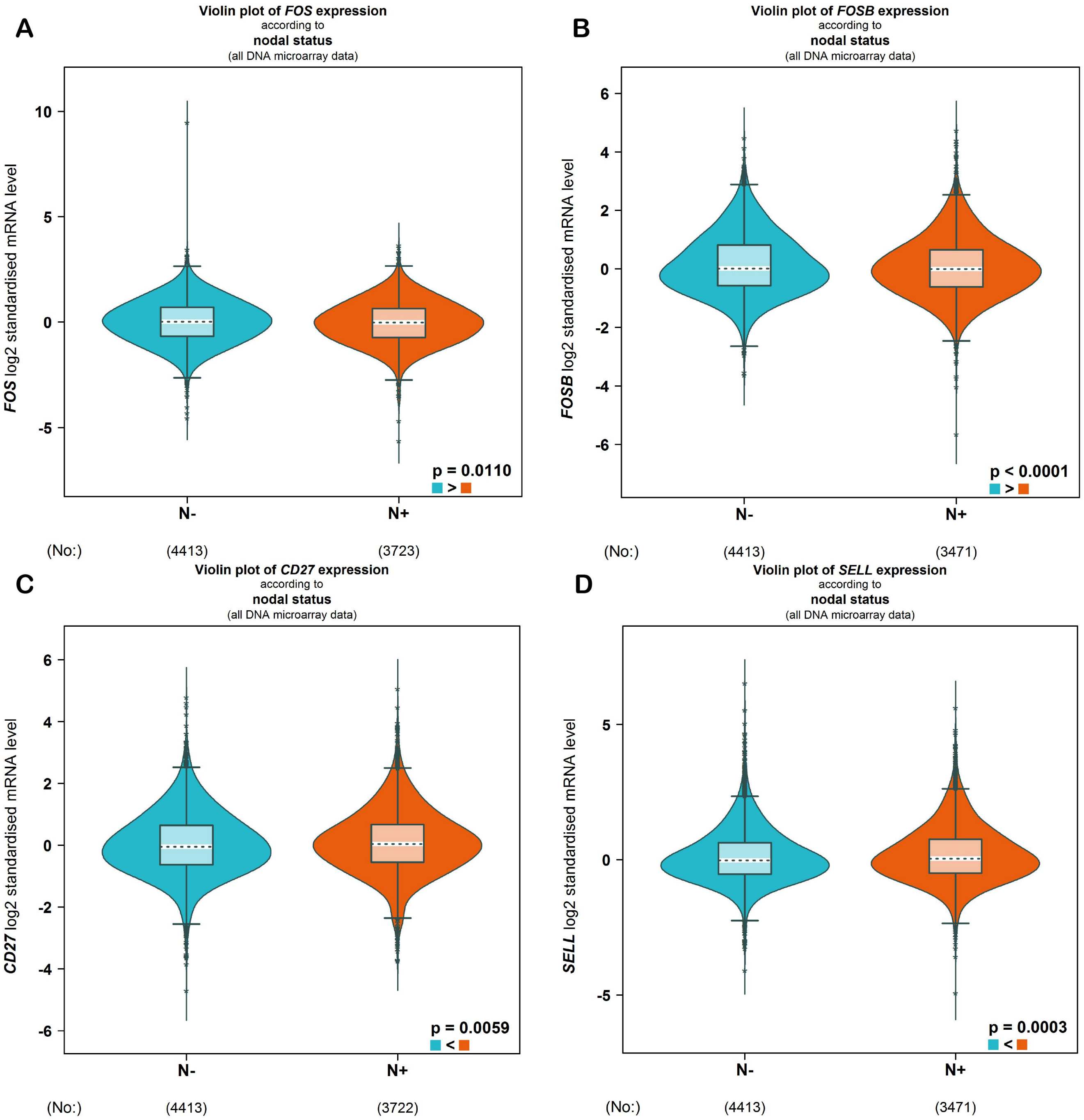

Figure S6. Expression levels of candidate genes based on nodal status of BC patients. Violin plots with p value < 0.05 are presented, as well as the corresponding p value.

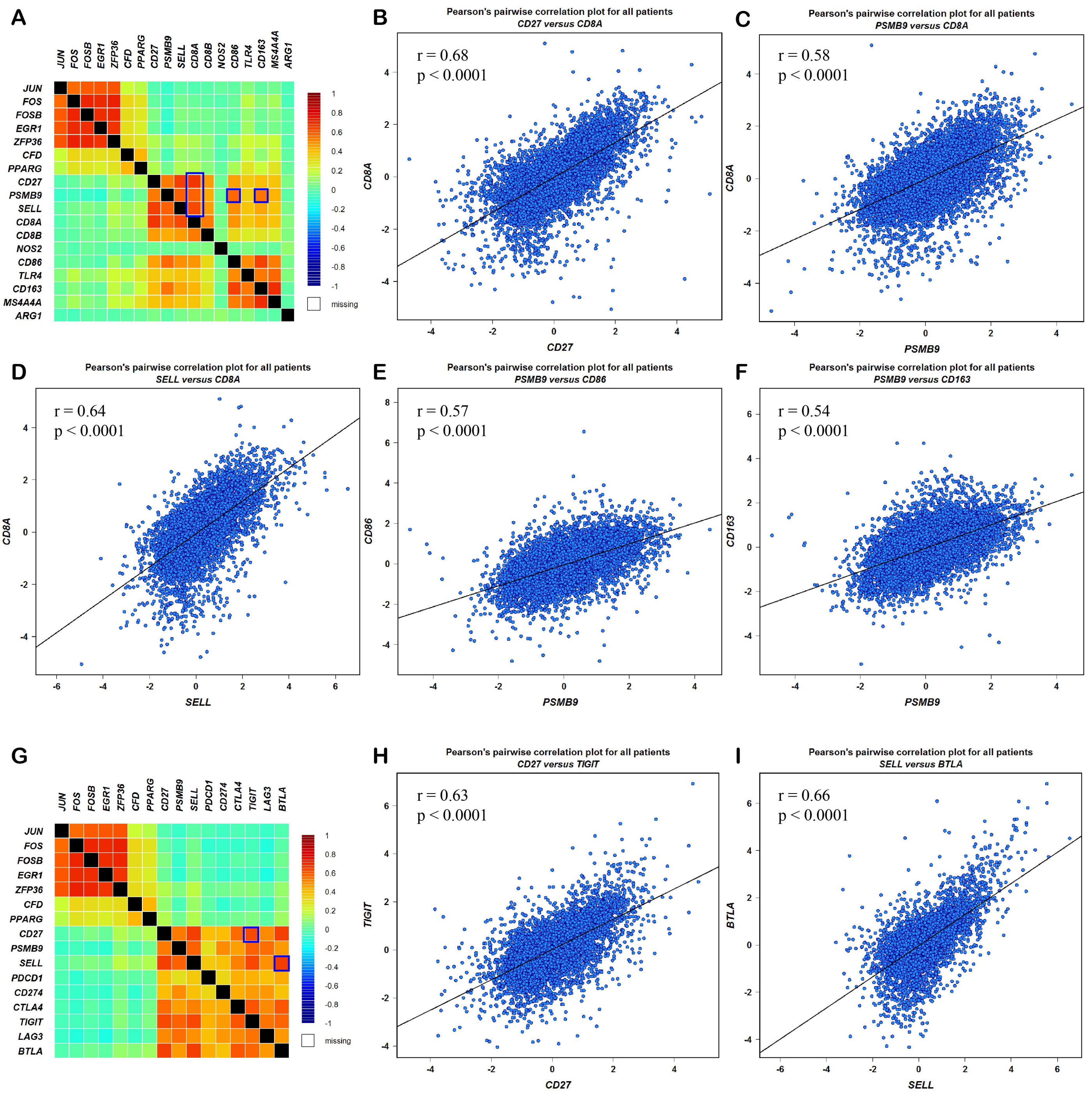

Figure S7. Expression correlations of candidate genes with biomarkers of immune cells and immune checkpoints. (**A-F**) Expression correlations of candidate genes with biomarkers of immune cells (CD8+ T cell, M1 macrophage and M2 macrophage). (**G-I**) Expression correlations of candidate genes with immune checkpoints.

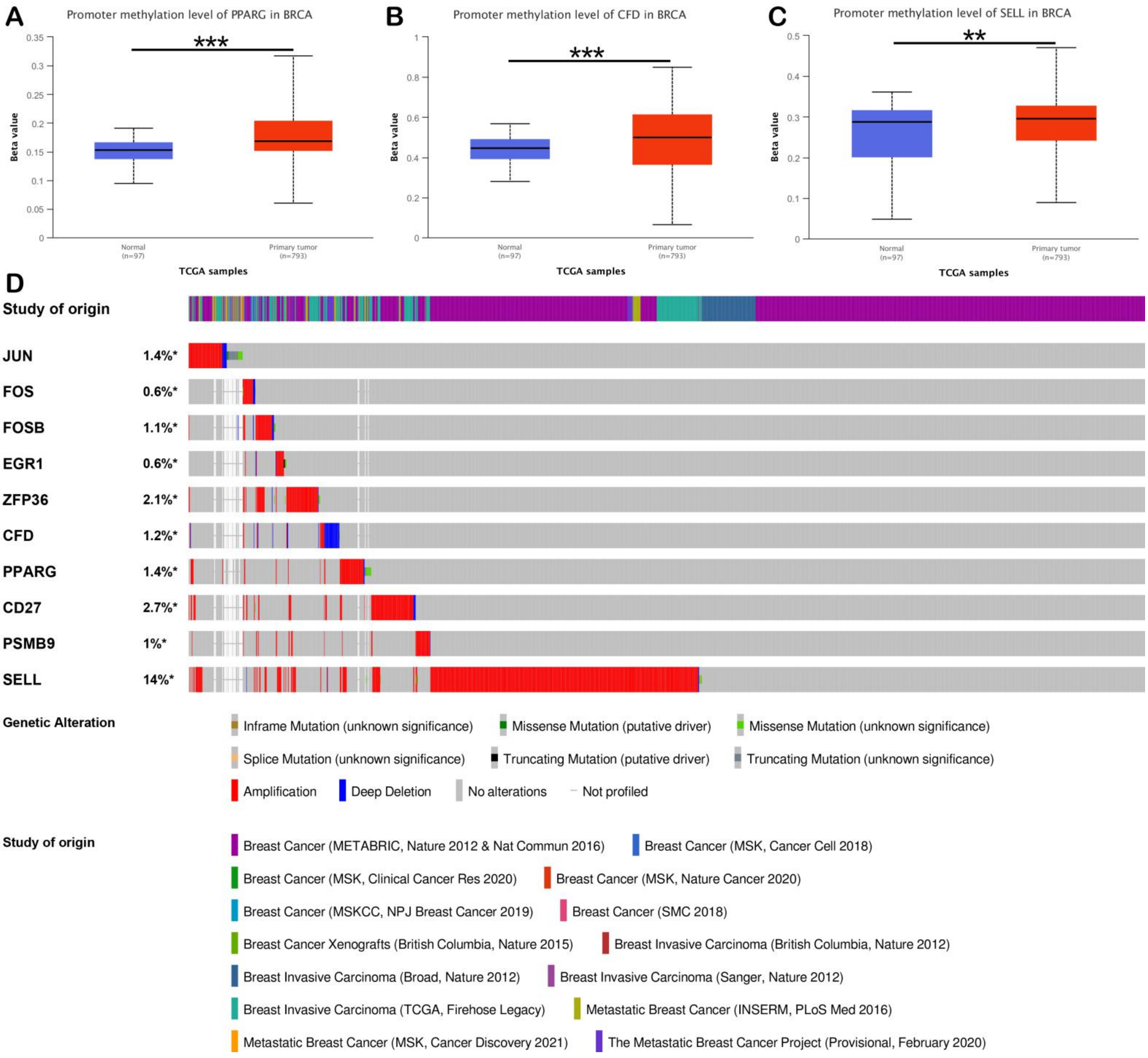

Figure S8. Promoter methylation and genetic alteration analysis for candidate genes. (A-C) Promoter methylation status of PPARG, CFD and SELL. **p < 0.01, ***p < 0.001. (D) genetic alterations of candidate genes.

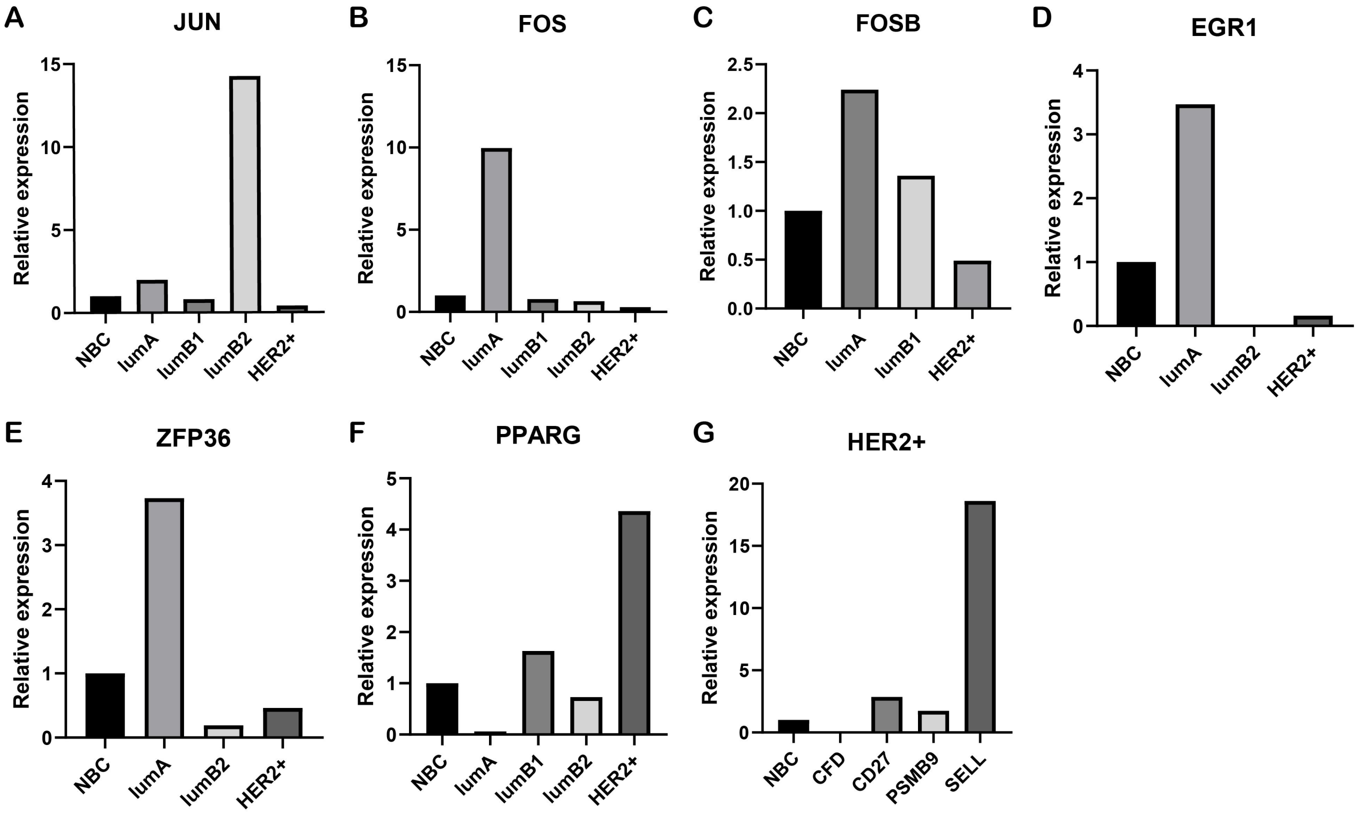

Figure S9. Relative expression of candidate genes in BC compared to pair normal breast tissues.

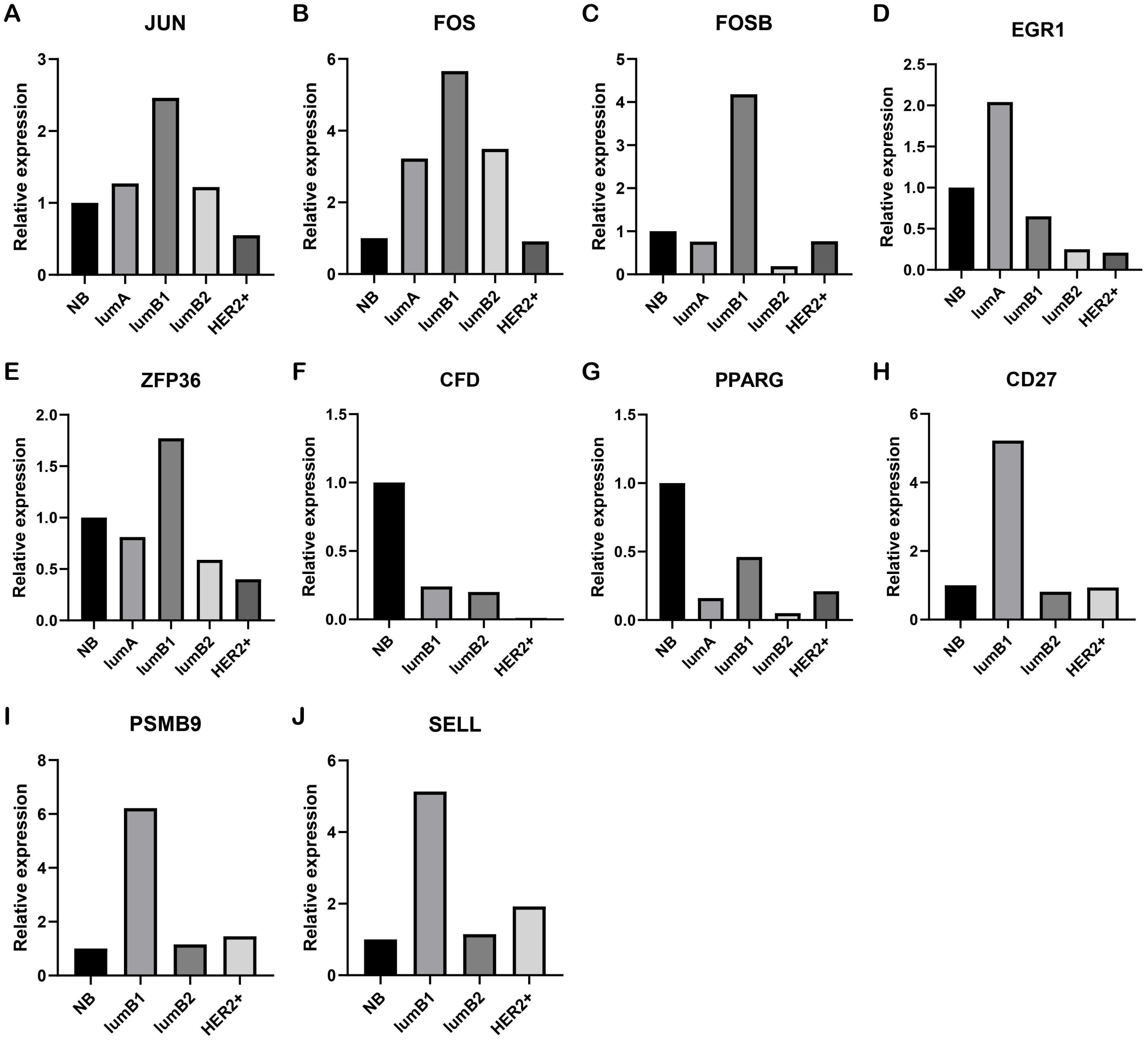

Figure S10. Relative expression of candidate genes in BC tissues obtained from 4 BC patients and non-BC tissues obtained from patients with benign breast disease.

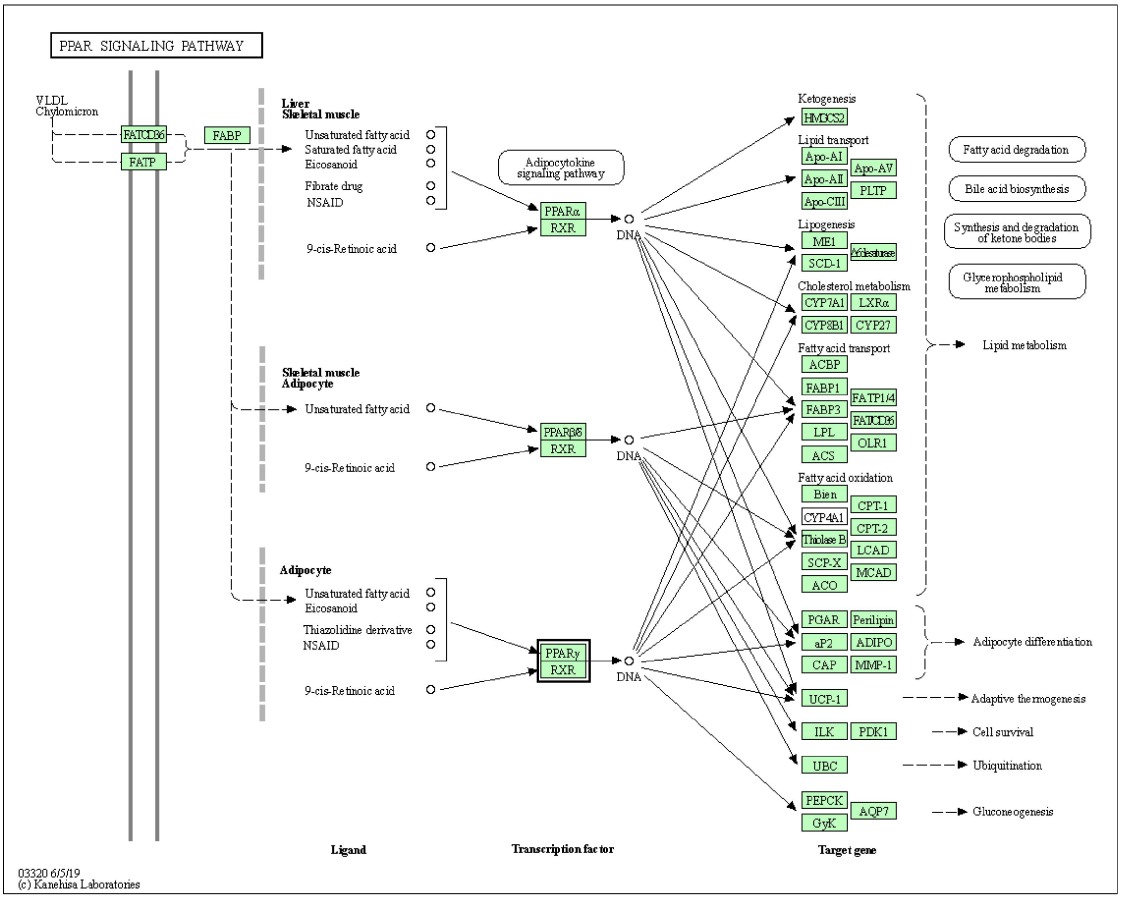

Figure S11. PPAR signaling pathway significantly enriched by DEGs of module 1 in GSE15852 dataset. PPAR gamma (i.e., PPARG) is stood out around by black box.

Table S1. Primer sequences of candidate genes used for RT-PCR in the present study

Candidate gene	Primer Sequence
JUN	Forward 5’-GCCAACTCATGCTAACGCAG-3’<br>Reverse 5’-CTCTCCGTCGCAACTTGTCA-3’
FOS	Forward 5’-GGGAGGACCTTATCTGTGCG-3’<br>Reverse 5’-ACACACTCCATGCGTTTTGC-3’
FOSB	Forward 5’-AGCAGCAGCTAAATGCAGGA-3’<br>Reverse 5’-CCACCAGCACAAACTCCAGA-3’
EGR1	Forward 5’-CCCCGACTACCTGTTTCCAC-3’<br>Reverse 5’-GACAGAGGGGTTAGCGAAGG-3’
ZFP36	Forward 5’-CACTCCTATCAGCGTCTGGG-3’<br>Reverse 5’-TCGAAGACGGGAGAGTCTG-3’
CFD	Forward 5’-TCAGCCACAGCGGCTTC-3’<br>Reverse 5’-GATCCGACCACGGGGC-3’
PPARG	Forward 5’-CCAGAAGCCTGCATTTCTGC-3’<br>Reverse 5’-TGGCATCTCTGTGTCAACCA-3’
CD27	Forward 5’-ATCAGCAACTGGGCACAGAA-3’<br>Reverse 5’-TGAGCCCAGTAGTGCCTCTC-3’
PSMB9	Forward 5’-CGCTTCACCACAGACGCTAT-3’<br>Reverse 5’-TGCCCAAGATGACTCGATGG-3’
SELL	Forward 5’-AGCACAGCACACTCCCTTTG-3’<br>Reverse 5’-TCATGGCTTTGCTTGGTCCT-3’

Table S2. Enrichment analysis of module genes detected from GSE9574 using Metascape web

GO	Category	Description	Count	%	Log10(P)
M167	Canonical Pathways	PID AP1 PATHWAY	5	62.50	-11.34
ko05166	KEGG Pathway	HTLV - I infection	5	62.50	-8.48
GO:0009991	GO Biological Processes	response to extracellular stimulus	5	62.50	-6.97

Table S3. Enrichment analysis of module genes detected from GSE15852 using Metascape web

GO	Category	Description	Count	%	Log10(P)
WP236	WikiPathways	Adipogenesis	8	50.00	-14.68
GO:0001101	GO Biological Processes	response to acid chemical	7	43.75	-9.59
GO:0010876	GO Biological Processes	lipid localization	7	43.75	-8.75
GO:0043627	GO Biological Processes	response to estrogen	4	25.00	-7.18
GO:0022407	GO Biological Processes	regulation of cell-cell adhesion	6	37.50	-7.01
GO:2000696	GO Biological Processes	regulation of epithelial cell differentiation involved in kidney development	3	18.75	-6.84
GO:0055088	GO Biological Processes	lipid homeostasis	4	25.00	-5.74
WP4321	WikiPathways	Thermogenesis	3	18.75	-4.53
GO:0048863	GO Biological Processes	stem cell differentiation	3	18.75	-3.41

Table S4. Enrichment analysis of module genes detected from GSE42568 using Metascape web

GO	Category	Description	Count	%	Log10(P)
R-HSA-198933	Reactome Gene Sets	Immunoregulatory interactions between a Lymphoid and a non-Lymphoid cell	7	24.14	-10.21
GO:0046649	GO Biological Processes	lymphocyte activation	11	37.93	-9.97
WP4884	WikiPathways	Pathogenesis of SARS-CoV-2 Mediated by nsp9-nsp10 Complex	4	13.79	-8.18
GO:0001817	GO Biological Processes	regulation of cytokine production	7	24.14	-4.90
GO:0032615	GO Biological Processes	interleukin-12 production	3	10.34	-4.49
GO:0030101	GO Biological Processes	natural killer cell activation	3	10.34	-4.04
GO:0009615	GO Biological Processes	response to virus	4	13.79	-3.32
GO:0038061	GO Biological Processes	NIK/NF-kappaB signaling	3	10.34	-3.02

Table S5. Validation of module genes applying UALCAN web based on TCGA and survival analysis using bc-GenExMiner resource

Datasets	Genes	BC vs. N		OS		DMFS		DFS	
		Log$_2$FC	Direction	HR (95%CI)	P value	HR (95%CI)	P value	HR (95%CI)	P value
	JUN	-1.16***	Down***	0.89(0.82-0.97)	0.0082**	0.92(0.83-1.01)	0.0689	0.91(0.85-0.97)	0.0059**
	FOS	-2.43***	Down***	0.77(0.71–0.84)	<0.0001***	0.75(0.68-0.82)	<0.0001***	0.79(0.74-0.84)	<0.0001***
	FOSB	-2.80***	Down***	0.81(0.74-0.88)	<0.0001***	0.79(0.72-0.87)	<0.0001***	0.84(0.78-0.90)	<0.0001***
	EGR1	-1.31***	Down***	0.85(0.78-0.93)	0.0002***	0.82(0.75-0.90)	<0.0001***	0.85(0.79-0.91)	<0.0001***
GSE9574	*IER2*	-1.48***	Down***	0.92(0.84-1.00)	0.0570	-	-	-	-
	NR4A2	-1.87***	Down***	1.00(0.91-1.09)	0.9210	-	-	-	-
	ZFP36	-1.52***	Down***	0.86(0.79-0.93)	0.0004***	0.84(0.76-0.92)	0.0002***	0.88(0.82-0.94)	<0.0001***
	ATF3	-2.31***	Down***	0.92(0.84-1.00)	0.0554	-	-	-	-
	ACSL1	-1.42***	Down***	1.10(1.01-1.20)	0.0244*	1.05(0.96-1.15)	0.2856	1.09(1.02-1.16)	0.0135*
	PCK1	-3.24***	Down***	0.93(0.85-1.02)	0.1138	-	-	-	-
	CIDEC	-1.14***	Down***	0.91(0.83-1.00)	0.0570	-	-	-	-
	RBP4	-1.14***	Down***	0.94(0.86-1.02)	0.1611	-	-	-	-
	CFD	-1.57***	Down***	0.86(0.79-0.94)	0.0006***	0.76(0.69-0.83)	<0.0001***	0.86(0.81-0.92)	<0.0001***
	LEP	-1.08***	Down***	0.85(0.78-0.93)	0.0005***	0.94(0.85-1.03)	0.1647	0.90(0.85-0.97)	0.0037**
	LPL	-1.75***	Down***	0.88(0.8-0.96)	0.0045**	0.93(0.85-1.02)	0.1222	0.94(0.88-1.01)	0.0724
	FABP4	-1.96***	Down***	0.85(0.78-0L93)	0.0003***	0.88(0.81-0.97)	0.0093**	0.91(0.85-0.98)	0.0071**
	PLIN1	-1.79***	Down***	0.87(0.79-0.95)	0.0022**	0.81(0.74-0.89)	<0.0001***	0.87(0.81-0.93)	<0.0001***
	ADIPOQ	-2.25***	Down***	0.85(0.74-0.98)	0.0235*	0.88(0.79-0.99)	0.0351*	0.92(0.85-1.00)	0.0520
GSE15258	*PPARG*	-1.01***	Down***	0.83(0.76-0.90)	<0.0001***	0.86(0.78-0.95)	0.0018**	0.87(0.81-0.93)	<0.0001***
	CD24	1.43***	Up***	1.21(1.10-1.32)	<0.0001***	1.22(1.11-1.34)	<0.0001***	1.13(1.06-1.21)	0.0005***
	KRT18	1.08***	Up***	1.19(1.08-1.30)	0.0002***	1.03(0.94-1.14)	0.4869	1.11(1.03-1.19)	0.0032**
	KRT19	1.87***	Up***	1.10(1.01-1.20)	0.0287*	0.96(0.87-1.05)	0.3758	1.04(0.97-1.11)	0.2477
	EPCAM	1.20***	Up***	1.16(1.07-1.27)	0.0005***	1.25(1.14-1.37)	<0.0001***	1.17(1.10-1.25)	<0.0001***
	GATA3	1.07***	Up***	0.85(0.78-0.92)	0.0001***	0.76(0.69-0.83)	<0.0001***	0.86(0.81-0.92)	<0.0001***
	PVRIG	1.17***	Up***	0.89(0.81-0.97)	0.0080**	0.92(0.83-1.01)	0.0900	0.88(0.82-0.94)	0.0001***
	CD27	1.95***	Up***	0.89(0.81-0.97)	0.0089**	0.90(0.82-0.99)	0.0253*	0.86(0.81-0.92)	<0.0001***
	IL21R	1.18***	Up***	1.01(0.92-1.10)	0.8824	-	-	-	-
	TIGIT	1.19***	Up***	0.99(0.89-1.09)	0.7712	-	-		
	MAP4K1	1.56***	Up***	0.98(0.90-1.06)	0.5672	-	-	-	-
	PTPRCAP	1.29***	Up***	1.00(0.91-1.10)	0.9986	-	-	-	-
	CCR7	2.12***	Up***	0.93(0.85-1.01)	0.1030	-	-	-	-
	HLA-F	1.37***	Up***	1.06(0.97-1.15)	0.2082	-	-		
	HLA-J	1.21***	Down***	-	-	-	-	-	-
	HLA-C	1.01***	Up***	1.06(0.97-1.16)	0.2055	-	-		
	HLA-B	1.06***	Up***	1.06(0.97-1.16)	0.2080	-	-		
GSE42568	*PSMB9*	1.20***	Up***	1.11(1.02-1.22)	0.0185*	1.07(0.97-1.18)	0.1707	1.04(0.97-1.11)	0.3196
	GPD1	-2.85***	Down***	0.86(0.79-0.94)	0.0007***	0.83(0.76-0.92)	0.0002***	0.86(0.81-0.92)	<0.0001***
	RBP4	-4.30***	Down***	0.94(0.86-1.02)	0.1611	-	-	-	-
	CIDEC	-5.35***	Down***	0.91(0.83-1.00)	0.0570	-	-	-	-
	PLIN4	-5.31***	Down***	0.85(0.77-0.93)	0.0005***	0.79(0.70-0.90)	0.0002***	0.88(0.82-0.95)	0.0017**
	PLIN1	-5.97***	Down***	0.87(0.79-0.95)	0.0022**	0.81(0.74-0.89)	<0.0001***	0.87(0.81-0.93)	<0.0001***
	CMPK2	2.44***	Up***	1.17(1.07-1.28)	0.0007***	1.12(1.00-1.26)	0.0469*	1.13(1.05-1.22)	0.0013**
	RSAD2	1.78***	Up***	1.19(1.09-1.31)	0.0002***	1.24(1.13-1.37)	<0.0001***	1.15(1.07-1.24)	<0.0001***
	MX1	1.98***	Up***	1.27(1.17-1.39)	<0.0001***	1.26(1.14-1.38))	<0.0001***	1.18(1.11-1.26)	<0.0001***
	IFI44	1.64***	Up***	1.11(1.02-1.21)	0.0166*	1.14(1.03-1.25)	0.0080**	1.09(1.02-1.16)	0.0125*
	IFI44L	1.19*	Up***	1.16(1.07-1.27)	0.0007***	1.20(1.09-1.32)	0.0002***	1.10(1.03-1.18)	0.0035**

CD2	1.56***	Up***	0.94(0.86-1.02)	0.1551	-	-		
SELL	1.34**	Up***	0.91(0.84-0.99)	0.0322*	0.93(0.84-1.02)	0.1141	0.90(0.84-0.96)	0.0021**
NLRC3	1.38***	Down**	-	-	-	-		
CD247	1.32***	Down**	-	-	-	-		
CD3G	1.52***	Down**	-	-	-	-		
EOMES	1.59***	Up***	0.94(0.86-1.03)	0.1905	-	-		
CD8A	1.34***	Down***	-	-	-	-		

$^{***}p < 0.0001$, $^{**}p < 0.01$, $^{*}p < 0.05$. HR, hazard ratio; CI, confidence interval. The genes highlighted in red were failed with validation. The genes highlighted in blue were the overlapping genes.

Table S6. The information and sample size of TCGA BC datasets

Characteristics	Living(N=683)	Deceased(N=106)	Total(N=788)	p-value
Stage				2.00E-08
I	123(15.71%)	15(1.92%)	138(17.62%)	
II	418(53.38%)	46(5.87%)	464(59.26%)	
III	139(17.75%)	34(4.34%)	173(22.09%)	
IV	2(0.26%)	6(0.77%)	8(1.02%)	
Age				
Mean ± SD	57.07±12.68	60.97±14.75	57.59±13.04	
Median [min, max]	56.00[27.00,90.00]	61.00[26.00,90.00]	57.00[26.00,90.00]	
ER				0.48
Negative	151(20.30%)	27(3.63%)	178(23.92%)	
Positive	494(66.40%)	72(9.68%)	566(76.08%)	
HER2				0.78
Negative	537(71.60%)	79(10.53%)	616(82.13%)	
Positive	115(15.33%)	19(2.53%)	134(17.87%)	
PR				0.82
Negative	210(28.26%)	34(4.58%)	244(32.84%)	
Positive	434(58.41%)	65(8.75%)	499(67.16%)	
Subtypes				0.53
Normal-like	14(2.34%)	5(0.83%)	19(3.17%)	
Luminal A	261(43.57%)	42(7.01%)	303(50.58%)	
Luminal B	105(17.53%)	22(3.67%)	127(21.20%)	
HER2+	35(5.84%)	8(1.34%)	43(7.18%)	
Basal-like	92(15.36%)	15(2.50%)	107(17.86%)	
Tumor				4.70E-06
T1	185(23.51%)	27(3.43%)	212(26.94%)	
T2	410(52.10%)	53(6.73%)	463(58.83%)	
T3	76(9.66%)	14(1.78%)	90(11.44%)	
T4	11(1.40%)	11(1.40%)	22(2.80%)	
Node				0.01
N0	346(44.13%)	36(4.59%)	382(48.72%)	
N1	221(28.19%)	45(5.74%)	266(33.93%)	
N2	73(9.31%)	18(2.30%)	91(11.61%)	
N3	39(4.97%)	6(0.77%)	45(5.74%)	
Metastasis				3.20E-08
Negative	593(85.45%)	91(13.11%)	684(98.56%)	
Positive	2(0.29%)	8(1.15%)	10(1.44%)	

TP53				0.37
Non-mutated	459(58.17%)	66(8.37%)	525(66.54%)	
Mutated	224(28.39%)	40(5.07%)	264(33.46%)	

Table S7. Univariate OS analysis using Cox Proportional Hazards model carried out by R with TCGA BC datasets

Variables	Univariate analysis		Variables	Univariate analysis	
	**HR (95%CI)**	**P-value**		**HR (95%CI)**	**P-value**
Stage	2.2 (1.6-2.9)	2.3e-07***	*PLIN1*	0.93 (0.86-0.99)	0.033*
Age	1 (1-1.1)	3.3e-06***	*ADIPOQ*	0.94 (0.9-0.99)	0.025*
ER	0.88 (0.56-1.4)	0.59	*PPARG*	0.8 (0.69-0.94)	0.0054**
HER2	1.3 (0.8-2.2)	0.27	*CD24*	1.2 (1.1-1.3)	0.0042**
PR	1 (0.66-1.5)	0.98	*KRT18*	0.99 (0.84-1.2)	0.92
Subtypes	0.97 (0.82-1.1)	0.73	*KRT19*	0.91 (0.8-1)	0.13
Tumor	1.5 (1.2-1.9)	8e-04***	*EPCAM*	1.1 (0.91-1.4)	0.26
Node	1.5 (1.2-1.9)	0.00015***	*GATA3*	0.98 (0.9-1.1)	0.7
Metastasis	4 (1.8-8.5)	0.00045***	*PVRIG*	0.74 (0.64-0.87)	0.00019***
TP53	1.1 (0.76-1.7)	0.55	*CD27*	0.79 (0.71-0.88)	1.5e-05***
JUN	0.77 (0.64-0.93)	0.0062**	*PSMB9*	0.83 (0.71-0.96)	0.014*
FOS	0.9 (0.81-1)	0.047*	*GPD1*	0.9 (0.83-0.96)	0.0032**
FOSB	0.91 (0.83-0.99)	0.037*	*PLIN4*	0.9 (0.84-0.97)	0.0063**
EGR1	0.89 (0.79-1)	0.068	*CMPK2*	1 (0.89-1.2)	0.79
ZFP36	0.83 (0.7-0.97)	0.023*	*RSAD2*	1 (0.9-1.1)	0.84
ACSL1	1.1 (0.95-1.3)	0.17	*MX1*	1 (0.89-1.1)	0.99
CFD	0.89 (0.8-0.98)	0.02*	*IFI44*	0.96 (0.84-1.1)	0.58
LEP	0.92 (0.85-0.99)	0.018*	*IFI44L*	0.98 (0.87-1.1)	0.68
LPL	0.92 (0.82-1)	0.15	*SELL*	0.81 (0.72-0.91)	0.00045***
FABP4	0.94 (0.89-1)	0.054			

*p<0.05, **p<0.01, ***p<0.001. HR, hazard ratio; CI, confidence interval.

Table S8. Multivariate OS analysis using Cox Proportional Hazards model carried out by R with TCGA BC datasets

Variables	Multivariate analysis for *CFD*		Variables	Multivariate analysis for *PPARG*		Variables	Multivariate analysis for *CD27*	
	**HR (95%CI)**	**P-value**		**HR (95%CI)**	**P-value**		**HR (95%CI)**	**P-value**
Stage	1.99 (0.90-4.41)	0.0902	Stage	2.05 (0.92-4.57)	0.0804	Stage	2.24 (1.02-4.93)	0.0454*
Age	1.05 (1.03-1.07)	2.99e-07***	Age	1.05 (1.03-1.07)	8.07e-07***	Age	1.05 (1.03-1.07)	5.94e-07***
ER	0.94 (0.40-2.25)	0.8937	ER	0.96 (0.40-2.33)	0.9334	ER	0.75 (0.32-1.74)	0.4979
HER2	1.53 (0.83-2.80)	0.1711	HER2	1.61 (0.88-2.96)	0.1243	HER2	1.69 (0.91-3.13)	0.0956
PR	0.86 (0.38-1.93)	0.7092	PR	0.82 (0.37-1.83)	0.6263	PR	0.78 (0.35-1.75)	0.5538
Subtypes	0.80 (0.58-1.11)	0.1819	Subtypes	0.82 (0.60-1.13)	0.2323	Subtypes	0.84 (0.61-1.15)	0.2686
Tumor	0.86 (0.54-1.34)	0.4971	Tumor	0.86 (0.55-1.36)	0.5272	Tumor	0.77 (0.49-1.19)	0.2355
Node	1.11 (0.90-1.77)	0.6715	Node	1.09 (0.68-1.74)	0.7354	Node	1.13 (0.71-1.80)	0.5996
Metastasis	1.66 (0.50-5.47)	0.4086	Metastasis	1.41 (0.42-4.77)	0.5801	Metastasis	1.64 (0.50-5.33)	0.4143
TP53	1.18 (0.62-2.25)	0.6205	*TP53*	1.29 (0.68-2.47)	0.4341	*TP53*	1.44 (0.74-2.81)	0.2811
CFD	0.84 (0.72-0.99)	0.0327*	*PPARG*	0.80 (0.65-0.998)	0.0478*	*CD27*	0.75 (0.66-0.87)	0.0001***

Variables	Multivariate analysis for *PSMB9*		Variables	Multivariate analysis for *SELL*	
	**HR (95%CI)**	**P-value**		**HR (95%CI)**	**P-value**
Stage	2.02 (0.93-4.40)	0.07579	Stage	2.22 (0.99-4.95)	2.3e-07***
Age	1.05 (1.03-1.07)	1.93e-07***	Age	1.05 (1.03-1.07)	3.3e-06***
ER	0.83 (0.36-1.95)	0.67639	ER	0.72 (0.30-1.71)	0.59
HER2	1.63 (0.88-3.03)	0.11948	HER2	1.80 (0.97-3.32)	0.27
PR	0.95 (0.42-2.18)	0.90841	PR	0.75 (0.33-1.70)	0.98
Subtypes	0.94 (0.68-1.30)	70748	Subtypes	0.78 (0.56-1.08)	0.73
Tumor	0.91 (0.59-1.41)	0.67753	Tumor	0.87 (0.55-1.37)	8e-04***
Node	1.07 (0.68-1.68)	0.78444	Node	1.07 (0.68-1.70)	0.00015***
Metastasis	1.87 (0.57-6.10)	0.29838	Metastasis	1.21 (0.36-4.08)	0.00045***
TP53	1.37 (0.70-2.69)	0.36242	*TP53*	1.48 (0.76-2.90)	0.55
PSMB9	0.73 (0.60-0.90)	0.00299**	*SELL*	0.77(0.66-0.90)	0.0062**

*p<0.05, **p<0.01, ***p<0.001. HR, hazard ratio; CI, confidence interval.

Table S9. GSEA according to the expression level of *FOS* and gene sets of KEGG pathway.

Datasets	KEGG pathways	SIZE	NES	FDR q-value
GSE9574	Positively correlated pathways			
	1. LEISHMANIA_INFECTION	67	1.80	0.125
	2. FC_GAMMA_R_MEDIATED_PHAGOCYTOSIS	87	1.71	0.215
	3. GRAFT_VERSUS_HOST_DISEASE	35	1.67	0.223
	4. ADIPOCYTOKINE_SIGNALING_PATHWAY	62	1.66	0.174
	5. ALLOGRAFT_REJECTION	33	1.64	0.182
	6. TYPE_I_DIABETES_MELLITUS	39	1.63	0.167
	7. VIBRIO_CHOLERAE_INFECTION	46	1.61	0.168
	8. ANTIGEN_PROCESSING_AND_PRESENTATION	73	1.56	0.239
	9. GLYCOSPHINGOLIPID_BIOSYNTHESIS_LACTO_AND_NEOLACTO_SERIES	23	1.56	0.214
	Negatively correlated pathways			
	1. LINOLEIC_ACID_METABOLISM	22	-1.73	0.140
GSE15852	Positively correlated pathways			
	1. FOCAL_ADHESION	187	1.64	0.198
	2. RETINOL_METABOLISM	38	1.64	0.232
GSE42568	Positively correlated pathways			
	1. FOCAL_ADHESION	194	1.93	0.023
	2. ADHERENS_JUNCTION	66	1.83	0.074
	3. COLORECTAL_CANCER	61	1.83	0.049
	4. ECM_RECEPTOR_INTERACTION	81	1.80	0.061
	5. TGF_BETA_SIGNALING_PATHWAY	80	1.75	0.101
	6. COMPLEMENT_AND_COAGULATION_CASCADES	67	1.70	0.154
	7. PATHWAYS_IN_CANCER	317	1.68	0.165
	8. WNT_SIGNALING_PATHWAY	146	1.65	0.200
	9. VALINE_LEUCINE_AND_ISOLEUCINE_DEGRADATION	43	1.65	0.181
	10. LYSOSOME	116	1.64	0.180
	11. FATTY_ACID_METABOLISM	40	1.64	0.167
	12. ACUTE_MYELOID_LEUKEMIA	56	1.60	0.211
	13. ENDOCYTOSIS	170	1.58	0.230
	14. AXON_GUIDANCE	128	1.57	0.236
